# Quantifying near-symmetric molecular distortion using symmetry-coordinate structural decomposition†

**DOI:** 10.1039/d4sc01670j

**Published:** 2024-08-06

**Authors:** Christopher J. Kingsbury, Mathias O. Senge

**Affiliations:** a School of Chemistry, Chair of Organic Chemistry, Trinity College Dublin, The University of Dublin, Trinity Biomedical Sciences Institute 152-160 Pearse Street Dublin D02R590 Ireland ckingsbury@ccdc.cam.ac.uk sengem@tcd.ie; b Institute for Advanced Study (TUM-IAS), Technical University of Munich Lichtenberg-Str. 2a 85748 Garching Germany mathias.senge@tum.de

## Abstract

We imagine molecules to be perfect, but rigidified units can be designed to bend from their ideal shape, discarding their symmetric elements as they progress through vibrations and larger, more permanent distortions. The shape of molecules is either simulated or measured by crystallography and strongly affects chemical properties but, beyond an image or tabulation of atom-to-atom distances, little is often discussed of the accessed conformation. We have simplified the process of shape quantification across multiple molecular types with a new web-accessible program – SCSD – through which a molecular subunit possessing near-symmetry can be dissected into symmetry coordinates with ease. This parameterization allows a common set of numbers for comparing and understanding molecular shape, and is a simple method for database analysis; this program is available at https://www.kingsbury.id.au/scsd.

## Abstract 2

A new method for analyzing crystal structures of molecules distorted from their regular shape separates atom movements into symmetry modes so that molecular conformation of functional materials can be quantified and compared easily across databases.

## Graphical abstract

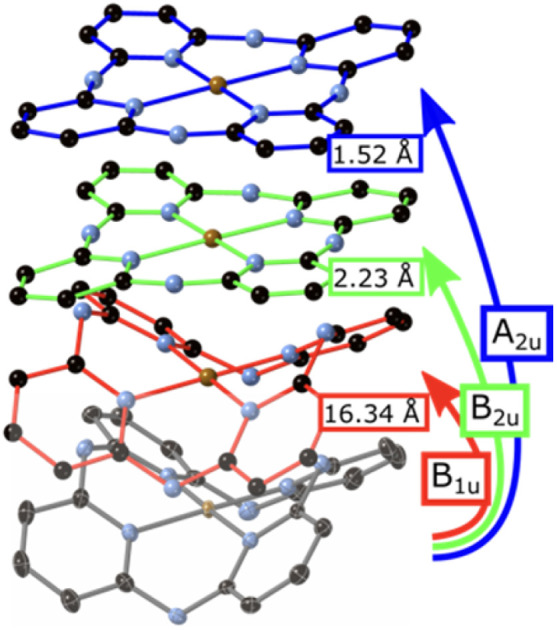

## Introduction

To understand a molecule, we must understand its shape. Chemistry, after all, is the study of nuclei and electrons, with electronic interactions and energy levels impacted by residence within molecular geometries. This is especially true of molecular components or rigid groups which are distorted from their usual arrangement, exhibiting stretching, twisting or bending from regular structure.^1,2^ Those molecules exhibiting irregular shape have distinct chemical properties from their regular counterparts, especially in interaction with light and with reaction partners. In crystal structure reports, distortion-induced change is often represented in qualitative terms which are difficult to compare across multiple structures.

Shape change in metal ion coordination environments is well known to affect measured properties, especially spectral, magnetic, and biological properties.^3^ Interaction with visible light is conceptually linked to d-block metal complexes and polycyclic, predominantly carbon molecules with distributed π-systems, each with electronic states separated by an energy compatible with the incident photon. First-row transition metal complexes are often three-dimensional, six-coordinate species near to the ideal octahedral coordination environment, with a history of numeric analysis derived from ligand effects and corresponding alteration of coordination environment, such as Jahn and Teller's work on spontaneous symmetry-breaking.^4^ Algorithms such as bond valence sum^5^ and the continuous symmetry measure^6^ allow for the digestion of the distortion of these coordination polyhedra into single numerical terms which assist the correlation of structure to properties. Symmetric-coordinate analysis as previously described by Murray-Rust, Bürgi and Dunitz^7^ for the simple enumerated inorganic polyhedra is perhaps the most sophisticated of these analyses, though has seemingly not found a place in routine crystal structure reporting. Small changes in inorganic polyhedra, especially in crystalline minerals, can be crucial to understanding photochemical properties.

Polycyclic aromatic molecules are often similarly approximately symmetric, though with far greater chemical diversity lack a similar common coordinate set which can be referenced. In aromatic molecules, chemical and electronic modification, steric demand and mechanical manipulation can be used to disrupt planarity, or indeed any of the symmetry elements; this distortion is usually described in qualitative terms.^8^ The quantification of distortion usually relies on the reporting of individual bond torsions or deviations from mean planes, but these numbers can quickly become unwieldy in large examples or conflate multiple structural relationships.^9^ Normal-coordinate studies^10–12^ which require calculated symmetry-gated vector tables are not readily available for molecules which may only have a few reported examples. Similarly, results generated without shared coordinates and vector tables are difficult to compare. Conformation in the solid state can be a particularly confounding problem for *ab initio* analysis, even for small molecules, given the multiple demands that inform shape upon crystallization. A mechanism to relate chemical structure to measured atom positions in a way which is quantitative, extensible, and predictive – while still being generally approachable – would allow a shared, unambiguous language to discuss shape. We present our approach to that parameterization here.

**Fig. 1:**
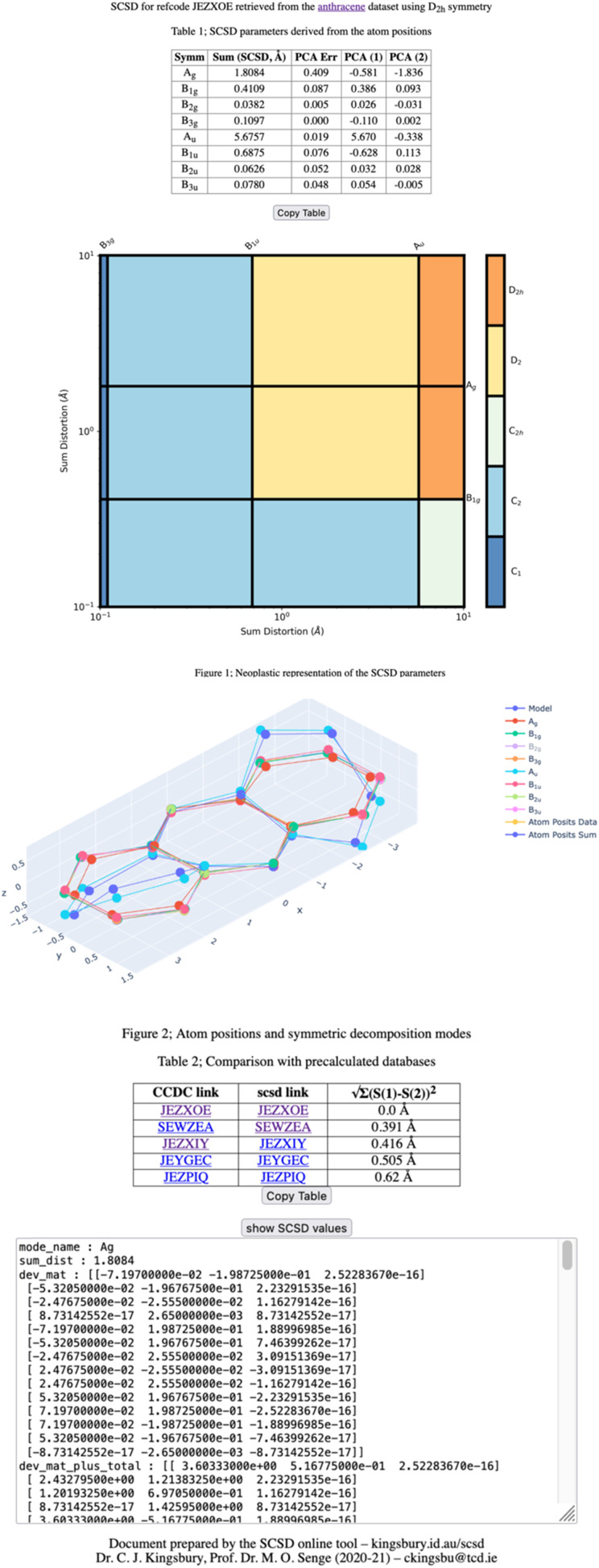
An example symmetry-coordinate structural decomposition output for a ‘twisted’ anthracene unit. The largest mode, A_u_, is the principal ‘bent’ shape, but small deviations from this *D*_2_ symmetry also contribute to the solid-state conformation.

Herein we present *Symmetry-Coordinate Structural Decomposition (SCSD)*, a program which decomposes 3D molecular coordinates into sets of symmetry coordinates, quantified and symmetry-delineated vectors in selected point groups (*C*_2v_, *C*_2h_, *D*_2h_ and *D*_4h_ among others). An example of the program output from crystallographic data of a substituted anthracene^13^ is provided in Fig. 1. The mathematics behind this decomposition are relatively simple – each symmetry-coordinate group is the application of the symmetry elements of an irreducible representation, and their sum is the original atom positions. Symmetry-coordinate analysis is not commonly encountered in routine structural studies, perhaps due to difficulty in access to programs. SCSD provides simple program access online as a narrative aid for individual structure reports that can be useful for understanding how and why a particular molecule may be distorted in a single crystal structure. As with Hirshfeld analysis for intermolecular interactions,^14^ or packing diagrams illustrating crystal lattices, a distortional profile quantifies organic molecules' accessed shape by simple inspection of a table or Mondrian symmetry plots,^15^ and allows discussion of shape in quantified terms.

## An example of SCSD analysis

Four outputs are returned to the investigator of a single structure, and these are shown in Fig. 1 for an exemplar non-planar aromatic – the anthracene core of 9,11,20,22-tetraphenyl-10,21-ditolyl-tetrabenzo(*a*,*c*,*l*,*n*)pentacene as the chloroform solvate (CSD: JEZXOE).^13^

The first table shows the magnitude of the symmetry-coordinate decomposition of the identified atoms. In this example, the twisted anthracene unit is most influenced by its A_u_ distortion, the largest of the deformations, as given by the “Sum (Å)” value. The A_u_ value is representative of the out-of-plane deformation which preserves the three *C*_2_ axes of symmetry of *D*_2h_, but not its other symmetry elements. This is determined by comparing the molecule positions with *C*_2_, 
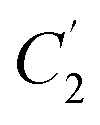
 and 
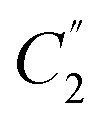
 symmetry operations applied, *versus* those with the *C*_2_, 
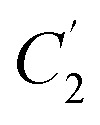
, 
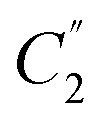
, *σ*_*xy*_, *σ*_*xz*_ and *σ*_*yz*_ operations applied (with A_g_ symmetry) to develop a vector set. The sum of the magnitudes of these vectors is a value of 5.68 Å, substantial for a small molecule and indicates a noticeable and persistent distortion to the molecular species.

A_u_ distortion is, however, only perpendicular to the mean plane of this species. The second-largest value of this column is the A_g_ distortion of 1.81 Å – encountered when comparing the A_g_ representation of the molecular unit with a model compound – anthracene itself. Bond distances are only altered by less than 0.04 Å; the majority of this distortion is an in-plane shadow of the A_u_ out-of-plane distortion. This is equivalent to how a right-angle triangle with a fixed hypotenuse will contract on the adjacent edge and increase on the opposite edge, as the angle is increased from zero. Additional distortional elements (B_1u_, B_1g,_ B_3g_) result from the crystal packing.

Principal component analysis (PCA) is explained in more detail below, but is simply a set of vector components which explain the most common deformation in molecules of each type, with each set of symmetry operations. As one can expect that molecules deform along their lowest-energy pathways, these resemble the vibrational normal modes which can be calculated for a small molecule but are derived from many crystal structures. Each of these normal modes is orthogonal to all other modes of that representation and can be fitted to our measurement data using linear summation; this table indicates that the first two principal components of A_u_ distortion (A_u1_: 5.67 Å, A_u2_: −0.34 Å), model this distortion with 99.6% accuracy (residual: 0.02 Å). Principal components can be explored in more detail through the ‘model’ page, which is accessible through the hyperlink at the head of the page.

The Mondrian diagram shows how out symmetry coordinates relate to the point group symmetry of the molecule.^15^ In any given crystal structure, atom positions will result from distortions from multiple intra- and inter-molecular forces at different magnitudes, and this diagram shows how these may relate to each other and the symmetry.

Large distortions will exert greater influence on properties and contribute more to our perception and narrative of the molecule in the solid state, and so influence more of the diagram's area. What most would recognize as the point-group symmetry of this molecule is an area center to upper-right of the diagram, a color region here indicative of *D*_2_ symmetry. The *C*_1_ symmetry imposed by the space group is shown in the lower left.

The third diagram is interactive and can be accessed online;^15^ with this viewer, one can explore in more detail the shapes resulting from symmetry-coordinate decomposition. An interactive 3D viewer is essential to understanding the shape of unfamiliar three-dimensional objects, and different modes can be shown or hidden by clicking on the legend.

A second table indicates the most similar conformations observed in the reference database, the CCDC CSD. These are calculated by minimum sum-square-distance of SCSD magnitudes and generally show compounds with similar chemistry, as well as shape. In this example, the most similar is the structure itself, then the same molecule as the ethyl acetate solvate, then three similar structures from the same paper.

More information is available in a click-box at the bottom of this page, including all the raw data that is used in the generation of the above plots. In all, these presentations show different aspects of molecular shape, and provide the investigator with quotable information on the deformation and symmetry.

## Program description

**Fig. 2:**
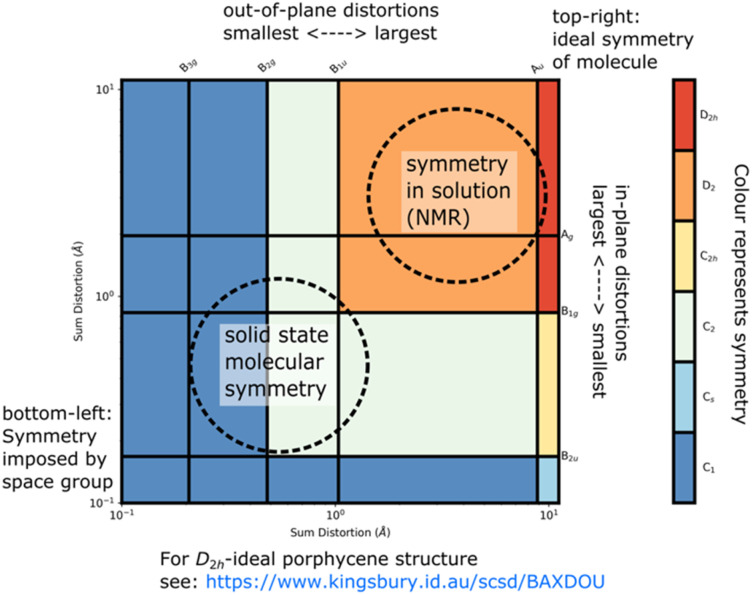
An annotated Mondrian symmetry diagram for an example saddle-shaped porphycene (CCDC: BAXDOU).^16^

A Symmetry-Coordinate Structural Decomposition (SCSD) of a set of molecular coordinates decomposes this model into a set of modes aligned to the irreducible representations of the point group. The sum of SCSD modes is identical to the input atom positions, whether from a single crystal X-ray diffraction (SC-XRD) experiment or simulation of molecular shape. These data can be presented as the simple magnitudes of these separated modes, the symmetry resulting from combined distortions as a Mondrian diagram (see Fig. 2),^16^ and an interactive visualization of these distortions in 3D space, each instructive in understanding accessed shape. From our previous studies on porphyrins,^12,15^ and for the examples in Table 1, we have found the magnitude (*e.g.*, B_2u_ > 6 Å) sufficient to indicate a shape, such as *saddle*, such that a name and number can be used mostly interchangeably.

**Table 1:** Exemplar pre-calculated molecular databases. All online available databases are listed at https://www.kingsbury.id.au/scsd_models_table

Molecular type	Name of example
Aromatics	Anthracene
	Pyrene
	Coronene^a^
Fluorophores	BODIPY
	Xanthene
Molecular electronics	Tetrathiafulvalene
	Tetracyano-α,α′-quinodimethane
	Dihydroxy-*p*-benzoquinone
	Naphthalenediimide
Porphyrinoids	Porphycene
	Corrole
	Norcorrole
	Corrphycene
	Chlorin
	Phthalocyanine
Other	Cucurbit[6]uril^a^
	Carbamazepine
	Salen
	β-Carotene
	Curcumin

a Additional approximations are used for non-perpendicular E-groups in *D*_6h_.

**Fig. 3:**
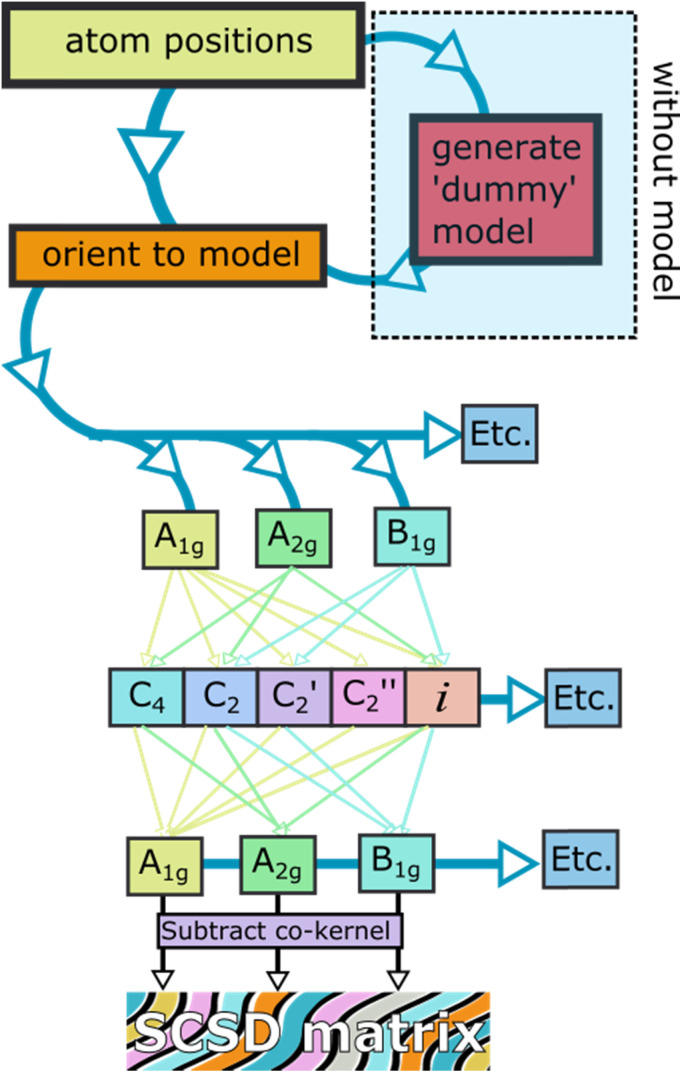
A flowchart showing the SCSD algorithm in *D*_4h_.

When smaller deformations appear, these may indicate favorable intermolecular interactions or vibrations from this principal shape. Data for case studies listed in Table 1 is available online,^17^ with data available from the authors. The SCSD program reports values as a real space magnitude, in Å, and can be reported and compared between series of similar molecules (for a flowchart of the procedure see Fig. 3). The magnitude is simply the summation of how much all atoms in the framework are deforming in a certain way. In more technical terms, this results in a set of vectors indicating deviation from co-kernel symmetry which obeys the operations of a given irreducible representation – one of the rows of a point group table. A larger number means more distortion away from a higher symmetry. Extracted coordinates can be used for further computational analysis through Principal Coordinate Analysis (PCA);^18^ this is best suited for comparison of large datasets. Individual structure analysis is designed to be as seamless to the investigator as possible, does not require any knowledge of the molecule except ideal point group symmetry and, optionally, a minimally distorted comparison example for determining totally-symmetric (A_g_, A_1_, A_1g_) distortion and faster computation.

Symmetry-coordinate analysis is performed in stages outlined in Fig. 3, and all are performed by the program automatically.

Firstly, the atoms and connections from the model are identified in the query structure and isolated, with the molecule moved and rotated to be a least-squares distance from the model. Secondly, the symmetry operations of each of the rows of the point group table are imposed on the atom positions separately, taking the mean of a position and its symmetry-equivalent atom yields coordinates which conform to that symmetry. For example, in *C*_2v_, we will generate four sets of coordinates, those corresponding to A_1_, A_2_, B_1_ and B_2_ – and these coordinate sets will have *C*_2v_, *C*_2_, *C*_s_(⊥*x*) and *C*_s_(⊥*y*) symmetry respectively. Thirdly, these coordinate sets are subtracted from their co-kernel symmetry, which is the next-most-symmetric representation of the molecule – in *C*_2v_, each of the co-kernels of A_2_, B_1_ and B_2_ is the A_1_ representation, and the A_1_ is compared to the model. This subtraction generates vectors which relate the model to its deviation in each symmetry representation, the decomposition; the summation of these decompositions is the original atom positions after the first step. Finally, the vector magnitudes are summed – this is what is reported as the symmetry-coordinate magnitude and can be understood as the amount to which the whole molecule is deformed in this manner.

Principal Component Analysis is an optional additional step for database analysis, in which vector sets from step 3 in multiple crystal structure collections are compared, with their most prominent common features identified. This generates a set of perpendicular vectors which are similar to normal modes, and a normal-coordinate decomposition can be performed. A tutorial is available at https://github.com/cjkingsbury/scsd/ that walks an investigator through the process.

Descriptions of similar algorithms have been reported which assess the ideal point-group assignment of calculated and experimentally-determined molecules^19^ – a similar process is used in SCSD to orient the molecule towards the relevant symmetry operations when no model is provided. SCSD analysis is conceptually the inverse of the continuous symmetry measure; splitting the positional data into constituent modes can allow each symmetry mode to be classed by its contribution to – rather than deviation from – any individual point group assignment.

The assignment of a value to the distortion of each molecule allows for structural chemistry databases to be interrogated as a function of shape, where similarly shaped molecules will presumably form clusters. This method allows more information to be determined with only a small number of measurements as orthogonal representations have minimal influence on one another. PCA, cluster identification and other methods to develop quantitative three-dimensional quantitative structure–activity relationships (3D-QSAR) are included in the library; analysis can be performed through a python notebook interface, such as Jupyter.^20^ Symmetry coordinate decomposition followed by PCA is found to be a good method of parameter reduction in structural chemistry, potentially useful for constraining crystallographic refinement. PCA is discussed further below.

Finally, this program addresses a niche for comparative structural analysis – simplifying (pseudo-)normal-coordinate analysis for a wide range of chemical substructures. Generalized structure prediction frameworks attempt to model structural variations while providing little in the way of insight, individualized structure description can be over-long and inconsistent between individual reports. We believe SCSD is suited for human-understandable structure comparison and a numerical approach to literature comparison in one publication, or to allow for insight and review of medium-sized sets (*ca.* 100–10 000 structures) derived from a crystallographic database. As such, one can succinctly summarize the conformational landscape of crystal structures of a single component. Our goals were easier generation of data, understanding and interpretation of shape. For this reason, we have provided an online interface – including interactive plots – so that the methodology is immediately understandable and within reach for all. Lastly, calculations can be performed in a notebook format; an easy and extensible procedure for many-structure investigation is included. SCSD can be installed with the command “pip install scsdpy”. This software is distributed under a licensing scheme that allows only non-commercial scientific and educational use.

With this program, we first sought to validate our assumptions around how molecules might deform from their symmetric state. From initial studies on exemplar molecules detailed in Table 1 (see ESI 2†), the first principal component mode describes 90–95% of the variance in the dataset, with the second PCA mode increasing this to ∼99%. Beyond the second PCA mode, residual distortion is normally below the usual atom positional accuracy in crystallographic analysis. A simplified model of distortion for a molecule [*e.g.*, boron dipyrromethene (BODIPY) distorts as *aileron* (A_2_) or *butterfly* (B_2_)] can be used as a convenient shorthand for the total distortion by symmetry arguments, for the first PCA mode A_2_(1), and for the first normal mode A_2_(1), all having similar shape. *Aileron* and *butterfly* are simple names that can relate these movements to familiar objects; equivalent designations like “twisting” or “folding” might be equally appropriate. No more than two parameters from each dissymmetric basis are required to generate a model as accurate as the measurement. This mirrors the way *saddle* and *ruffle* shapes (towards *D*_2d_) of porphyrin derivatives^9*a*,12,21^ have a semantic, approximate meaning coincident with the symmetry coordinates and a fixed normal coordinate vector description. Across the structures indicated in Table 1, no meaningful relationships were found from chemical patterning to molecular shape beyond the first two parameters of each irreducible representation, excepting A_1g_, A_1_ or A_g_. When different strategies are employed to distort a molecule – steric hindrance, fusion of an additional aromatic ring or exocyclic strapping, for example – new examples of molecules could still be modelled satisfactorily with combinations of these same two parameters. This finding is in line with our earlier findings on porphyrin macrocycles.^12^

Symmetry-coordinate magnitude is a simple, measured shape descriptor that aligns with how shape is often discussed in structural chemistry. This simple version can compare the structural features of a limited crystal series as the shape may change with, *e.g.*, temperature, pressure or in an electronic excited state. Structures with similar SCSD values will be similar, constrained by related chemical demands; similarly shaped molecules will hold similar SCSD values and display similar shape-determined properties. If many molecules with a certain chemical patterning access the same shape, it is reasonable to assume that this shape persists to the chemical in solution, for example.

Just as the distortion is important, so the symmetry that is accessed is an important feature. These symmetry coordinates are how a chemist may describe shape when wishing to relate that a structure is perhaps *both bent and twisted* – it is sometimes hard to relate that this combination results in *C*_2_ symmetry. The presence or absence of symmetry can affect the allowed or forbidden optical transitions, as in Laporte's rule.^4*d*^

Further investigation of a dataset requires that a set of normal modes is generated; these can be implemented from external sources, such as molecular mechanics simulation, or calculated from provided data through PCA. As such, this program can be used to ascertain the accessed conformational space which best approximates the data, with components suggestive of the calculated lowest frequency vibrational modes for a particular subset.

With these tools, data from thousands of structure reports can be decomposed using only a handful of expressions and summarized using these small parameter sets. Examples of structure comparison are provided below; databases and notebooks are available upon request. These data are an excellent beginning point for structural review of each individual molecular component in quantitative terms.

The SCSD tool (see ESI 1† for a description of the program) is available to use online at https://www.kingsbury.id.au/scsd, or as a standalone program for academic use through GitHub or by contacting the authors.^22^

## Discussion

The interest in writing an easy-to-use general form of normal-coordinate analysis came from our investigations surrounding the “Mondrian” symmetry graphs (see Fig. 2), in which the dissymmetric modes of a molecule are presented as a pseudo-artwork pastiche.^15^ Mondrian plots are a method which demonstrates the approximate molecular point group at multiple levels of scrutiny to the atom positions. Where a molecule appears, for example, in *Z*′ ≥ 1 structures, the molecule may have no symmetry which relates its constituent atoms; nonetheless, approximate symmetry relationships, and their measurement, are often readily observable. This plot style, described in detail in our previous paper displays the magnitude of symmetry coordinates on logarithmic axes as vertical and horizontal lines; these lines each act to separate two symmetry point groups – with, and without the consideration of that dissymmetric movement. As we progress from upper right to lower left, we cross more of these boundaries and take on more and finer-grained information of the molecule, and its distortional profile becoming ever closer to the measured. At the same time, we progress from an idealized representation to the measured one, often with *C*_1_ representation. In reverse, from lower left to upper right, we would be acquiring symmetry in the sequence of smallest distortions, conceptually making the molecule more symmetrical by removal of distortion until arriving at our idealized model. We intuit molecules as somewhere between these two extremes – the precisely measured “real” and the assumed perfect “forms”.

The emergent features of these graphs were intriguing, especially the L-shaped correlations between the in-plane and out-of-plane features, often observed for a porphyrin distorted from regular structure.^12,21^ Similarly, the regularity of logarithmic molecular distortion was both aesthetically pleasing and implied that there may be a deeper relationship between the elements of molecular structural distortion. As part of projects aimed at using conformational design for the molecular engineering of functional porphyrin scaffolds,^23^ we have investigated the structural underpinnings of porphyrin-based chiral sensors and organocatalysts,^24^ where large and small distortions from structure both play an important role. ‘Artworks’ such as the ‘Mondrian plots’ present a view into an obfuscated world of picometer-scale distortion and fine control, allowing a simple additive metric for understanding this molecular chirality. The additive nature is important, as any chiral photochemical or templating effect can be expected to grow in proportion to the dissymmetry imposed on the molecule.

Investigating the intricate relationships between solid-state structure, symmetry, and substitution, moving beyond porphyrins to apply general insights to molecules required these new tools. We found that, owing to the tendency for similar molecules to bend in similar ways, using a normal-coordinate framework and its underlying pre-computed modes was not necessary for generating meaningful insight from the artwork or when writing about shape. The general solution of point-group-derived symmetry coordinates was compelling as an analysis routine for individual structures as all it required was the atom positions.

Crystallography for small molecules generates a lot of precise data;^25^ how these molecules are reported is at the discretion of the crystallographer, relying on a wealth of experience in determining the important structural features and close contacts. In routine organic communications, however, often little space is reserved for discussing the molecular shape or packing beyond structure validation. The conformation of an individual molecular unit is perhaps of little interest to the synthetic chemist – modulated by the molecules' immediate crystal environment, polymorphism and the assumptions or ‘fixes’ made by the crystallographer in solving the structure, including space group, features not necessarily representative of the structure in solution. The influence of these constraints can be downplayed by looking at multiple structures of the same unit, understanding molecular shape through careful comparison of multiple samples of a conformational landscape; not usually pursued except where polymorphism is indicated. SCSD, intended as an extra analysis routine in the tool-belt of a crystallographer, allows one to rapidly assess, contextualize and compare varied crystallographic data on symmetry and concerted atom movement terms, and simplifies the process of normal-coordinate analysis. For example, where one would report a mean deviation from a plane (a convolution of any number of distortional types), one can report the components of distortion in irreducible representation terms. This interface is designed to function on an unmodified *.pdb* or *.sd* input file and can be performed and understood without any specialist knowledge in computational chemistry.

A semantic divide can be observed in the distortional modes – between those which are intended (or designed), and therefore contribute to a “canonical” symmetry representation (how the molecule may be described), and those deviations which are below this arbitrary threshold. Canonical representation is not something fixed; this may be a conformation profile consistent with spectroscopic results, observed in solution-state conformational studies or in most crystal structures, or simply a common conformational cliché common to a structure type. Smaller modes can be thought as components of a vibrational structure upon crystallization; while potentially useful in the assignment of vibrational and vibronic spectra, these also indicate how the conformational compromise to minimize lattice energy is achieved. These modes and deviances are typically ignored in structural discussion. Structural variance under polymorphism would indicate multiple local minima of this combined function – either small (in disorder) or large – as two or more distinct self-catalyzed precipitation processes, such as (2,3,7,8,12,13,17,18-octaethylporphyrinato)nickel(ii) – which exhibits distinctly different near-planar and ruffled polymorphic examples with the same chemical composition.^26^

**Fig. 4:**
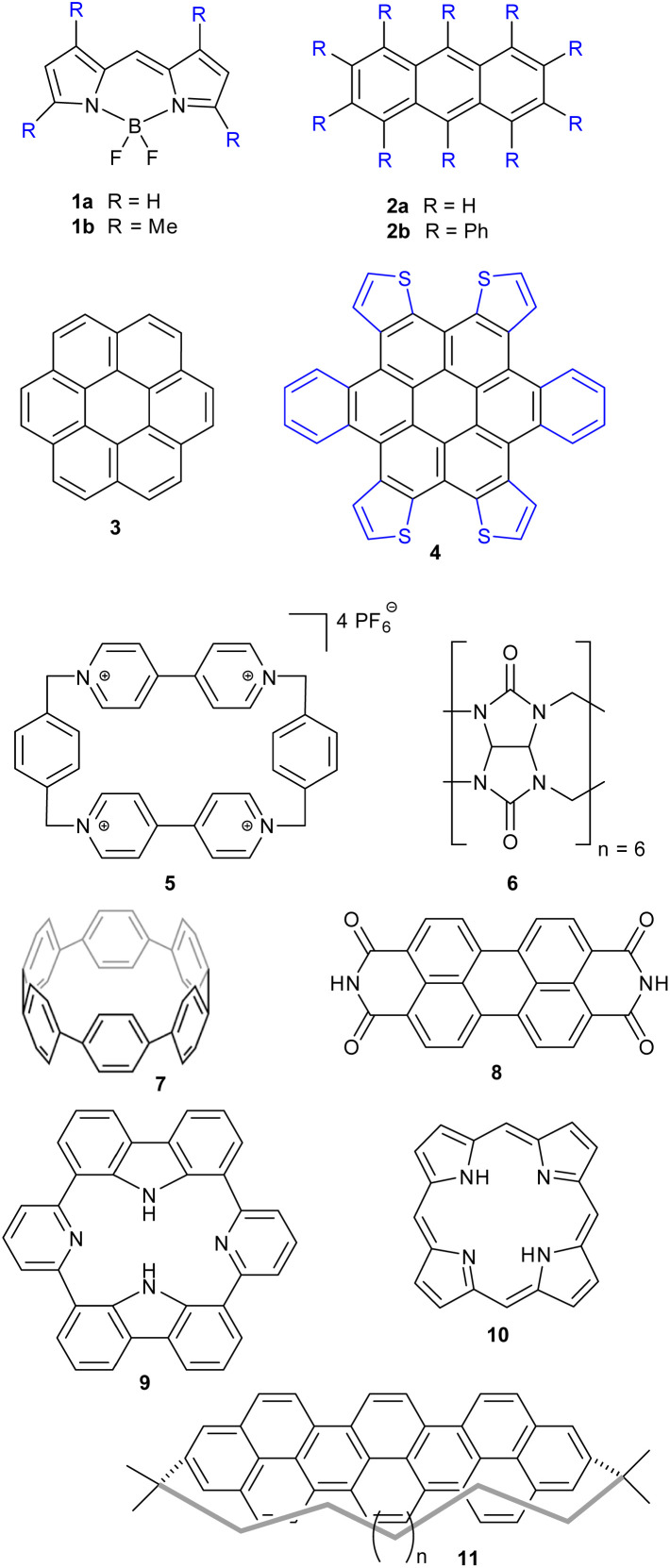
Examples of classes of compounds used for illustration.

SCSD-generated magnitudes can be used in a similar manner to how Normal-coordinate Structural Decomposition (NSD) has been used for porphyrin analysis – to determine the effectiveness of any distortion-inducement strategy, such as metalation, oxidation state change or peripheral substitution, and to correlate these strategies with photophysical attributes and biological functions in small datasets.^10–12,15,21,27^ With this program, a large database of conformational information can be generated for structural review and cluster analysis on any near-symmetric molecular subunit, an easy method of surveying the conformational landscape of a component, *e.g.*, those substructures indicated in Fig. 4. Comparing structures generated by screening methods or through serial crystallography often requires the selection of choice attributes of the crystal, like a select bond distance; symmetry coordinates are an easy, fast global parameterization, and normal coordinates are ideal for turning this insight into bidirectional quantitative relationships through curve fitting.

Normal-coordinate analysis is a worthwhile endeavor for the emerging field of automated and machine learning-driven solid-state analysis,^28^ allowing for parameter reduction while sacrificing little in the way of atom-positional accuracy. SCSD parameterization allows for symmetry modes to be derived directly from the symmetry-banded data, rather than from precalculated modes, and therefore removing at least one layer of computational model complexity. Similarly, compared with the simple direct PCA of crystallographic data, SCSD parameterization allows a significantly smaller data set to produce chemically meaningful results. Using refinement constrained to these measured parameters, more accurate solutions to poor quality data from, *e.g.*, cryo-electron microscopy might be made, or approximation of solution state structure by analysis of symmetric concerns independent of the constraints of crystallographic confinement, from spectroscopy.^29^

Machine learning approaches to chemical reasoning (*e.g.* ANI-1)^30^ can be incredibly useful for understanding interaction and conformation of individual units, though may fail to accurately gauge aromaticity or interaction profiles and tend to exclude metal centers. We believe the questions of interest to organic and coordination chemists, however, are often much smaller in scope – usually constrained to only one structure type, and deeply rooted in similar structure comparison rather than global modelling. SCSD allows one to reduce a query on distortional predilection imposed by a particular substitution pattern on a shared, symmetrical frame to a statistical relationship. All tools required to make those assessments are included, with this runtime efficient enough to run on laptop hardware in minutes for large data sets.

**Fig. 5:**
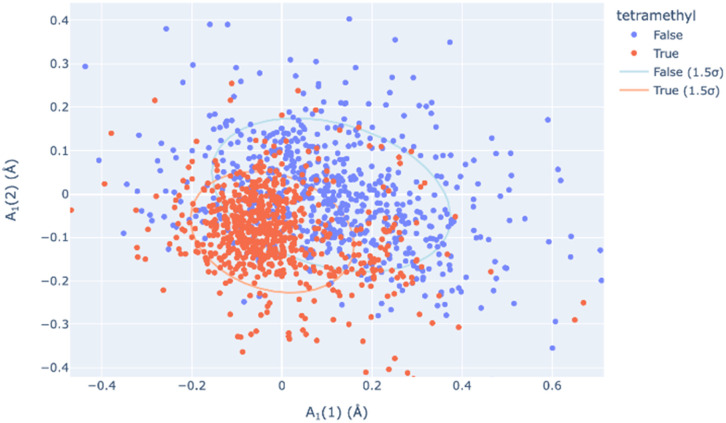
The A_1_(1) and A_1_(2) principal components of structural decomposition in BODIPY compounds (1) – two distinct but overlapping clusters of symmetry coordinate vectors represent those with a 1,3,5,7-tetramethyl substructure (1b, blue) from those without this substructure, including 1a (red).

Principal Component Analysis, from scikit-learn and implemented by this module, allows one to determine the best-fitting vector set for a large database of symmetry-banded vectors, showing similarity to the low-frequency vibrational modes which can be calculated. PCA can be a method of relating the distortional magnitude to atom positions bidirectionally. SCSD PCA is included in *scsd.py*; this taps a rich vein of structural insight when considering multiple structures, allowing the investigator a method of clustering and sampling similar molecular shapes. For example, in a comparison of BODIPY (boron dipyrromethene) molecular compounds (1),^31^ substitution of a 1,3,5,7-tetramethyl-moiety (1b) may seem to have little effect on the structure compared to the unsubstituted comparators (Fig. 4). However, these two motifs can be separated into two clusters by a two-component model in A_1_ symmetry of the BODIPY core (Fig. 5 and ESI 3.1†). Real insight can be gained by numerical analysis of many structures, and, hopefully, these tools bring that analysis into the realm of the non-specialist. Should we subscribe to the axiom that each of the crystallographically-determined positions for a given component are a near-random sampling of the vibrations accessed by the canonical structure, these principal components accurately represent the measured vibrational structure of molecules, something unattainable by direct measurement previously. In some irreducible representations, symmetric molecular components exhibit a clear signal for spontaneous dissymmetry; investigation of this behavior is ongoing. Space group symmetry can clash with approximate point group near-symmetry of crystallographically-inequivalent fragments; the constraints imposed upon measurement should be considered in the reporting of these values, especially those that would impose strict coplanarity of components, and corresponding zero SCSD values.

The point groups available in the first iteration of the SCSD algorithm are *C*_2v_, *C*_2h_, *D*_2d_, *D*_2h_, *D*_3h_, *D*_4h_ and *D*_6h_, covering common multiple-symmetry chromophore molecules of interest. As for simpler cases, in *C*_1_, this algorithm becomes a simple sum of vectors between an aligned fragment and model. With only one non-identity symmetry operation, the trivial case result is analogous to an implementation of the “symmetrize” routine and the “*S*” value of the parent symmetry.^19^ While these structures are possible to probe with the SCSD routine, we believe the utility of this routine comes from disentangling multiple modes, and more conventional comparisons may be more appropriate in these cases.

A Mondrian-style diagrammatic representation^15^ of these symmetry concerns can illuminate synchronistic dissymmetry by the same methodology presented previously for porphyrin molecules; these are generated as part of the SCSD runtime by use of a predetermined symmetry lookup table. These diagrams are an elegant manner of presenting symmetric information at multiple levels, as a symmetry mode is clearly visible both in magnitude and effect on the total symmetry of the idealized molecule. From here, choice of what is considered ‘important’ symmetry is left to the investigator.

## Examples of SCSD analysis

We have endeavored here to give useful and instructive examples of the analysis of distorted molecules across different areas of chemistry, mostly planting the idea of what can be found using these investigative tools (see ESI 3† for full descriptions). Of course, these examples are not exhaustive, nor is SCSD limited to just these frameworks.

Anthracene (2a) is a very common motif in organic chemistry. The distortion from regular planar structure resulting from decaphenyl – (2b)^32^ or similar substitution around the anthracene periphery has A_u_ character – and predictably yields a near-*D*_2_ conformation where the aromatic frame is twisted (ESI 3.2†).^32,33^ Other types of side groups (*e.g.*, sulfur decoration) or other distortion-inducing patterns (alkyl strapping) intermolecular interactions, chemical modifications or inclusions in large cycles, can influence atom displacement in distinct ways, each with a characteristic SCSD footprint. A researcher may compare their novel determination with all anthracene-containing structures in the CCDC-CSD^25^ to determine most-similar structures, for example, or to draw a substitution-distortion relationship.

Larger aromatics, such as substituted coronene (3) materials, are useful as model compounds for graphene, as well as nonplanar polycyclic aromatic hydrocarbons being interesting molecular electronics components. As an example, *a*,*d*,*j*,*m*-tetrathienyl-dibenzocoronene (4) has *D*_2h_ symmetry on paper – steric clash between adjacent units bifurcate the molecular modelling into two distinct conformations, with *C*_2h_ and *C*_2v_ near-symmetry (ESI 3.3†).^34^

**Fig. 6:**
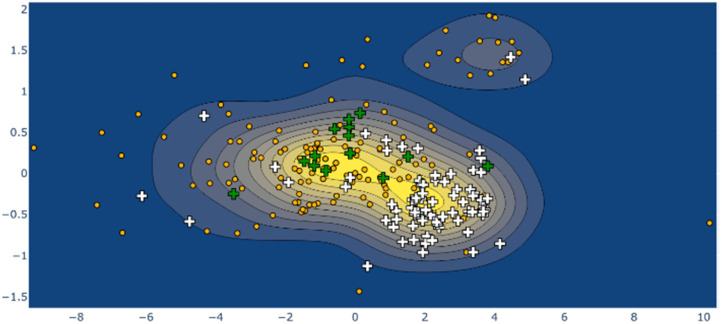
KDE plot of the ‘blue box’ A_g_ distortion; naphthalene-containing structures are shown as white crosses, tetrathiafulvalene-containing structures as green crosses, other structures as orange dots. The ‘diradical’ structure cluster is shown in the upper right of frame.

Larger still, a molecular fragment such as Stoddart's “Blue Box” (5),^35^ a mainstay of host–guest chemistry, can be quantified for its response upon inclusion of other fragments. It is an important early example of supramolecular design, and how molecular machines can be controlled by redox state. The cycle exists as a strained near-*D*_2h_ symmetry compound and has 225 examples in the CSD. One obvious separate cluster can be identified in the data of the first two A_g_ principal components, where a distinct cluster of data points (the radical, reduced form) is evident from the kernel density estimation (KDE, Fig. 6, upper right), as well as a bisected main cluster [naphthalene-including and non-naphthalene (TTF, hydroquinone) including]. This is one method of showing the clear structural distinction of host–guest chemistry on the molecular structure. Clearly, the structural variation from different inclusion compounds, chemical structures and crystallization conditions introduces noise, but clear signals can be obtained, and features identified and summarized succinctly. Because these derived parameters are not dependent on any individual atom, this can be a robust method of assigning oxidation state in organic molecules.

Even larger groups, such as the cucurbit[6]uril toroidal host (6),^36^ can be studied in this automatic fashion, illuminating the mechanisms of host response towards different intercalants (ESI 3.5†). *E.g.*, threading of an alkyl chain promotes expansion; with NH_2_ units, as in 1,5-diaminopentane, a contraction can be observed. A flat aryl group can be seen to promote an elliptical deformation.

Other test cases reported in the ESI† are for cycloparaphenylenes (*e.g.*, 7) as nanotube fragments and example of structures with rigidification by angular strain (ESI 3.6†);^37^ perylene-3,4,9,10-tetracarboxylic diimide (8, PDI) (ESI 3.7†), esp. distorted PDI compounds as circularly polarized luminescent materials;^38^ dipyridyldicarbazoles (9) as model compounds for investigating shape-assisted self-assembly (ESI 3.8†);^39^ a selection (ESI 3.9†) of porphyrinoids (porphyrin 10) comprising Sessler's amethyrin as an example of expanded porphyrinoids,^40^ Brückner and Meyer's Siamese twin porphyrin,^41^ nonplanar phthalocyanines from Kobayashi's group,^42^ a triply-fused diporphyrin from Osuka and coworkers,^43^ and corroles.^44,45^ Other examples are a study of teropyrenes (11, ESI 3.10†) developed by Bodwell's group allowing assumptions about the likely shape of molecules in the series,^46^ and an analysis of the application of time-resolved crystallography to elucidate structural changes upon photoexcitation in inorganic complexes (ESI 3.11†).^47^ Lastly, a brief list on Mondrian^15^ types is given (ESI 4†).^48^

These individual data sets are all available online (https://www.kingsbury.id.au/scsd_models_table^49^) and at *e.g.*https://www.kingsbury.id.au/scsd_model_ext/subporphyrin, or by contacting the authors. A worked example of the analysis of a phthalocyanine molecule with a ruffle deformation (CSD: FOLDET)^50^ is provided in ESI 1.7;† an example of using a notebook interface is included in the module available from github.^51^

Different approaches are being explored to using this information in chemical and protein database analysis, serial crystallography interpolation with pressure and temperature variations and ultrafast X-ray free electron laser (XFEL) studies, for crystallographic data validation and error-checking. This algorithm is potentially applicable to all pseudo-rigid groups or near-symmetry molecules, as the program is designed to be chemistry-agnostic in this regard.

## Conclusions

The SCSD routine has been described and offers a method to quantify perturbations of simple molecular components using symmetry-specific vector sets. We have shown previously that distortional vectors encode important chemical and photophysical information, and here are demonstrated to act as a simple method of parameter reduction and fitting for molecular shapes adopted by near-symmetric molecules. A numerical structure description is of immense value to the crystallographer, assisting in understanding and relaying structural concepts across structural sets and series. The story that can be told with a small set of numerical values, in the same manner as a constructed set of normal-coordinate vectors, allows for the non-specialist to see deeper into the shape of molecules and place their shape in the proper context.

Assigning a fixed symmetry point group to a dynamic molecule is an oversimplification in many cases. SCSD is a method of quantifying that compromise between diagrammatic symmetry and measured positions in crystal structures. The notion of sum-of-vectors is a comfortable one for a chemical crystallographer, we believe, given familiarity with vibrational modes in physical chemistry. Extension of this parameterization proves just as effective in ascribing shape to simple aromatic systems, macrocycles and larger molecular groups as it has been for denoting the shape of coordination polyhedra. Finally, this is an efficient means of sampling an accessible conformational space from minimal observations, often simply those that have been determined by prior researchers.

We invite our colleagues to use SCSD terms in multi-structural analysis and review – this is a structural comparison technique which can yield genuine insight into chemical properties and the efficacy of strategies toward dissymmetry. Given that often individual crystallography papers can offer only limited context for their reported structures, a numerical basis allows one to formalize these insights succinctly and with statistical power. We hope that this conformational control, in concert with chemical modification, can elicit new modes of reactivity and tuning of the innate properties of organic and coordination compounds.

## Experimental section

The SCSD module is written to be compatible with python 3.8, with numpy,^52^ scipy, networkx, matplotlib^53^ and plotly^54^ libraries, using Flask^55^ for web templating. A copy of this program is available on request from the authors for academic use, and at https://github.com/cjkingsbury/scsd.^51^

The alignment of point-group symmetry operations with a cluster of points in three-dimensional space is made by a minimization of the sum distortion attributable to the various symmetry operations – a least sum of pairs of the vectors *V* and *V* × [*A*], representing the original and transformed, assigned structures. As any residual rotational and transformational displacement will contribute to dissymmetric distortion, this correct alignment of the atomic structure is important to attain consistent values. The existence of separated ‘basins’ in even simple analyses requires either a basin-hopping algorithm or choice of multiple perpendicular starting points – the use of six perpendicular orientations was found to be sufficient to achieve the global minima in most cases.

The use of a quaternion parameterization allows for an approximately uniform minimization space.^56^ The symmetric operations rotation, improper rotation, and mirror plane can be defined as operations on the unit sphere, by spherical coordinates (*ϕ* and *θ*) converted into the unit vector [*x*,*y*,*z*]; rotations are defined as a rotational unit quaternion [*x*,*y*,*z*,*w*] (*w* = 2/*n*) applied to the coordinates, mirror planes as twice the dot product of the initial coordinates and the vector [*x*,*y*,*z*] subtracted from the coordinate matrix, and an improper rotation as the sequential application of reflection and rotation; inversion is simply [[−*a*,−*b*,−*c*]⋯].^12^ Atoms are assigned to their symmetry-opposite equivalents by using a Hungarian method solution to the assignment problem.^57^ After alignment of the molecule to the symmetry operations, a dummy symmetric model is generated by iteratively averaging the atom positions with their symmetry equivalents through each operation of the point group; in non-2-fold axes, this is repeated. An assignment matrix for each of these operations is cached in a *scsd_model* object. This first step is skipped if a precomputed model is provided.

The query atoms are aligned by least-squares minimization or rotation and translation either to a predefined model or to this dummy model. A molecular graph-based method for the assignment of query atoms to the model is also available for very distorted structures (*by_graph*). There exists an upper limit to distortion where a structure with normal connectivity may be incorrectly assigned by the simple program, the *by_graph* option can assist some of these edge-cases.

Symmetric vectors attributable to each of the irreducible representations are determined by subtraction of the “symmetrized” molecule with the symmetry operations defined by one row of an expanded point group table (*i.e.*, without grouping of equivalent operations), from the co-kernel symmetric representation – the totally symmetric representation of the class in most cases. The totally symmetric query component (A_g_, A_1_, A_1g_, *etc.*) is compared to the model to attain an indication of the totally symmetric distortion, equal to zero when no model is used.

SCSD values obtained by this method are the sum magnitude of the individual displacement vectors which comprise the eigenmodes, equivalent to the sum of normal coordinates for a well-constructed set. Eigenmodes are shown in an interactive visualization, with details in the raw data output.

Formation of a symmetric, aligned model is performed at https://www.kingsbury.id.au/scsd_new_model, or equivalently by the *yield_model* subroutine in notebook interface, and is stored in *scsd_models_user.py*. This is the first step in generating a dataset; where axes are interchangeable, models are aligned such that variance var(*x*) > var(*y*) > var(*z*) for *D*_2h_, so that near-planar molecules will generally lie in the *x*–*y* plane, and var(*x*) > var(*y*) for *C*_2v_ (*x*–*z* plane); in this way, interconvertible mode descriptors are generally consistent between measurements. Symmetric models for the compounds in Table 1 have been pre-computed and can be used with the web interface to accelerate computation, and new models can be uploaded and used for individual structure comparison.

Some point-group specific concerns are necessary when dealing with E- or T-symmetric modes; for example, in *D*_4h_ the number of possible E_g_-mode orientations is 4, on *x*, *y*, *x* + *y* and *x*–*y* (2*C*′ and 2*C*′′) axes, where only two are necessary for a complete description of the system. The sum of these modes is multiplied by ½ to arrive at the correct atom positions, and the Mondrian diagram uses only the greatest magnitude vector and its orthogonal counterpart to describe the symmetry of the molecular component. This is achieved by using the derived eigenmodes as input vector sets in a *pseudo*-normal coordinate analysis, which is the *simple_scsd* output of the *scsd_matrix* object.

Model generation is the only computationally expensive step; large-scale database analysis is rapid even for large queries. Symmetry coordinate analysis becomes most useful for comparisons between large number of structures; this can be formulated by use of an extracted database (in .sd format) and a chosen model; the commands for dealing with large numbers of structures are contained within the *scsd_collection* object.

Installing the scsd module is most easily performed with *pip install scsdpy*. The simplest method of interaction is by using *scsd_mercury.py* from the program examples,^51^ through Mercury, which identifies those models which have been previously defined and runs a symmetry-coordinate analysis based on SMARTS matching of the substructure. Additional interfaces are available to perform database and multi-structure analysis.

## Data availability

The data supporting this article have been included as part of the ESI.† The SCSD tool is available to use online at https://www.kingsbury.id.au/scsd or as a standalone program for academic use through GitHub (https://github.com/cjkingsbury/scsd/).

## Author contributions

C. J. K.: conceptualization, investigation, methodology, software, formal analysis, data curation, writing – original draft, review & editing. M. O. S.: investigation, methodology, funding acquisition, resources, supervision, writing – original draft, review & editing.

## Conflicts of interest

There are no conflicts of interest to declare.

## Supplementary Material

SC-OLF-D4SC01670J-s001
