# Supplementary material for: Quantifying near-symmetric molecular distortion using symmetry-coordinate structural decomposition
Source: Chem Sci. 2024 Aug 6;15(34):13638–49. doi: 10.1039/d4sc01670j (PMC11310747; doi:10.1039/d4sc01670j)
Supplement: SC-015-D4SC01670J-s001 [file SC-015-D4SC01670J-s001.pdf]

Supplementary Information (SI) for Chemical Science.
This journal is © The Royal Society of Chemistry 2024

Supplementary Information

Table of Contents:

1.	The SCSD Program	2
1.1	General information	2
1.2	Using the SCSD package	2
1.3	Fitting an individual molecule	3
	GUI	3
1.4	Generation an .sd file for import using Mercury (4.0, with CSD-Materials)	3
1.5	Generating a database (scsd_collection object)	4
1.6	Generating a model (scsd_model object)	4
	GUI	4
1.7	A worked example of analyzing nonplanar distortion in phthalocyanine molecules	5
2.	Implemented Models	7
3.	Test Cases of SCSD Analysis	9
3.1	BODIPYs	9
3.2	Anthracene	9
3.3	Tetrathieno-dibenzocoronenes	11
3.4	The "blue box"	13
3.5	Cucurbituril CB[6]	13
3.6	Cycloparaphenylenes	14
3.7	Perylene-3,4,9,10-tetracarboxylic diimide	15
3.8	Dipyridyldicarbazole	16
3.9	A selection of porphyrinoids	17
3.10	Teropyreneophanes	19
3.11	Time-resolved crystallography – [Cu(I)(dmp)(dppe)][PF_6]	20
4.	Mondrian Typing	23

1. The SCSD Program

1.1 General information

The symmetry-coordinate structural decomposition module is written in python, and relies on the following libraries:

- Numpy – arrays, functions, constants, and linear algebra
- Scipy – minimization functions for global 2eople2ry2on and linear sum assignment
- Matplotlib – the 'surface' plot which underlies the Mondrian graph.
- Plotly – interactive plotting in the web-accessible environment
- Seaborn – sequential and named color maps
- Flask (jinja2) – the webserver components
- Pandas – storage and retrieval of databases relating multiple structures.
- Sklearn – Principal component analysis (PCA) to determine pseudo-normal modes.
- Networkx – graph-based molecular identification and fitting.

Generation commands are shown in the attached notebook.

1.2 Using the SCSD package

This is a package which contains several components for the symmetric decomposition of molecules or of databases of molecules. The general data structure is:

Main –

scsd.py	Main program, scsd_matrix and scsd_collection objects
scsd_models.py	defines *scsd_model* object, list of available models, coordinates etc.
scsd_symmetry.py	Defines scsd_symmetry, underlying assignment tables for select point groups
scsd_models_user.py	Extra models from the webserver interface
scsd_webserver.py	Webserver pages and methods – can be launched with:<br>python scsd_webserver.py<br>or using wsgi
ellipsoid.py	Methods for cluster identification
/data/scsd/	scsd dataframes containing reduced data
/data/temp/	Cache of .pdb files
/templates/scsd/	Templates for html pages

There are four objects:

- *scsd_matrix* – an individual structure's scsd values and methods
- *scsd_model* – the idealized model
- *scsd_collection* – for a database of many structures
- *scsd_symmetry* – underlying class that collects symmetry functions

These each have many subroutines to assist a single- or many-structure investigation. These are discussed in turn.

1.3 Fitting an individual molecule

GUI:

- Launch the SCSD webserver, or go to www.kingsbury.id.au/scsd
- Upload a .pdb file which contains either:
    - only the designated atoms; *i.e.,* for a bodipy, only the $C_9N_2BF_2$ core should be in the .pdb file.
      <u>or</u>
    - a small molecule structure which includes the atoms of a named fragment (e.g., “bodipy” in the above example) when using a model
- And use the rest of the form to indicate:
    - the ideal point-group (“C_{2v}”)
      <u>or</u>
    - a pregenerated model from the list below (“bodipy”)
- Optionally:
    - indicate a colormap. Try “Spectral” or “random”
    - add ‘basinhopping’ if the analysis has errors
    - add ‘by_graph’ if the molecule is very distorted from the model

Command line / notebook:
Have your atoms in a .pdb file, or in a numpy array of cartesian [[x,y,z,a]…] or [[x,y,z]…] (Å) where a are atom identifiers (e.g. “C”).
Use these commands:
> import scsd
> ats = scsd.import_pdb(‘/path/to/bodipy.pdb’)
Import the atoms from a pdb file.
> sc = scsd.scsd_matrix(ats, ‘C2v’, model = ‘bodipy’)
Run the calculation:
> sc.calc_scsd()

There are now a few available methods, most relating to the visualization of data. *Html_table*, *scsd_plotly, compare_table* and *raw_data* are the simple components which are shown in the web-accessible version; *dev_plot* and *dev_table* are deviation plots in cylindrical coordinates for macrocyclic compounds, similar to the implementation for porphyrins.

1.4 Generating an .sd file for import using Mercury (4.0, with CSD_Materials)

1. Load a query feature, a representative example of the class. Can be from a file or from the CSD.
2. Open Mercury and go to CSD-Materials > Search > Crystal Packing Feature.
3. Select the atoms of the query. Remember, these should be minimally distorted and be only those of the main fragment, with the appropriate symmetry (e.g. the aromatic fragment of something like pyrene)
4. Unclick ‘number of hydrogens’ but keep ‘number of bonds’ for non-metal-coordinated atoms. Next, Next –
5. Select the entire CCDC database (full geometric search, slow) or a previously identified subset from ConQuest, and any additional structures you’ve loaded, and move those to the right-hand screen. Next.
6. Select ‘3D coordinates determined’ and any other parameters you’d like to narrow the search.

7. Hit “search”
8. When finished “save results” as “Overlaid hit fragments (.sd)”

Note: a .sd file can be generated similarly from the protein data bank using their online interface; this data can be imported using this same procedure, or through passing a list of coordinates directly to scsd_collection from a compatible API.

1.5 Generating a database (scsd_collection object)

You’ll need: a model (See: *generating a model*)
An .sd database (see: *Generating an .sd file using Mercury*)
> import scsd, scsd_models
> bodipy_test_model = scsd_models.scsd_model(‘bodipy_test’,
scsd_model_objs.get(‘bodipy’).ats_4) # ‘bodipy’ is a built-in model
> bodipy_collection = scsd.scsd_collection(bodipy_test_model)
> bodipy_collection.sd_file_to_simple_df(‘path/to/file.sd’, Verbose = True, by_graph = True)
> pcas = bodipy_collection.gen_pca()
> bd_df = bodipy_collection.gen_complex_df()

If there are any errors in individual structure assignment (i.e. obvious outliers):
> for refcode in [‘FUBARR’,’MESTUP’,’BADSTR’]:
bodipy_collection.recalc_simple_df_row(refcode)
Your scsd_collection object is now set up properly to do your data science study- the available methods are *gen_web_df* and *write_df* which cache the database for later comparison through a webserver, and *plot_pca* to look at the principal component analysis results. Data are most easily accessed in the “complex_df” object, which is a Pandas DataFrame; plotting these variables with plotly.express can be performed easily.

1.6 Generating a Model (scsd_model object)

You will need a minimally distorted experimental or computationally minimized model of the atom positions of the isolated fragment of interest, in the .pdb format.
Using the GUI:

Generating a Model:

GUI:
- Launch the SCSD webserver, or go to www.kingsbury.id.au/scsd_new_model
- Upload a .pdb file which contains only the designated atoms.
- Indicate the ideal point-group (*i.e.,* “C_{2v}”) by radio button.
- Indicate the maximum distance of usual atoms. Default is 1.75.
- add ‘basinhopping’ if the analysis has misidentified the fragment.
- Add a name for interacting with this model in the future. This is saved in scsd_models_user.py and can be imported through scsd_models.model_objs_dict.get(‘name’)

The model can be copied from the “Show SCSD Values” box at the bottom of the model’s web page.

Command line / notebook:
Have your atoms in a .pdb file, or in a numpy array of cartesian [[x,y,z,a]…] or [[x,y,z]…] (Å) where a are atom identifiers (e.g. "C"). Use these commands:

```
> import scsd, scsd_models
> ats = scsd.import_pdb('/path/to/bodipy.pdb')
> ptgr = 'C2v'          # point group
> model_ats = scsd.yield_model(ats, ptgr)
> model = scsd_model('name', model_ats)
```

Your model object, once initialized, can have principal components added through the scsd_coll.*gen_pca()* routine. The model can be saved by running scsd_coll.*write_df()* (writes to scsd_models_user) or *model.importable().*

1.7 A worked example of analyzing nonplanar distortion in phthalocyanine molecules

In practice, the analysis may be performed by a scientist upon the collection of a crystal structure of a molecule – such as phthalocyanine (*Pc*) which is distorted from planarity (Kidd, S. R., Zhou, W., Warren, J. J., Leznoff, D. B., *Dalton Trans.*, **2024**, *53*, 6537, DOI: 10.1039/D4DT00528G). This paper demonstrates admirably how distinct deformations are very different in both instantiation and properties in its class, adapting an analyser we designed for the related porphyrin ring. The analysis presented below is intended to show how one may more easily investigate phthalocyanine natively without assumptions that may be inherited from the porphyrin-native approach. The crystal structure used in this example is phthalocyaninato-bis(2-(1,3-diisopropylimidazolyl))-iron (CSD: FOLDET).

Phthalocyanine is already a model listed in www.kingsbury.id.au/scsd_models_table, which makes the required data pre-treatment minimal. The phthalocyanine molecule is saved into a .pdb format using free software, such as Mercury, while ensuring that the entire molecule is included in the file – because we are using a pre-defined model, additional atoms do not have to be excised. This .pdb file is then uploaded into the form at www.kingsbury.id.au/scsd and "phthalocyanine" is typed into the box marked "Model". Finally, the "by_graph" option can be ticked – this simply speeds up analysis in most cases. The form is submitted – calculation should take fewer than 5 seconds.

The returned form is one which displays a table of scsd information, the Mondrian diagram (see Angew.Chem.) and an interactive three-dimensional display of the atom positions. Finally, a table indicates which similar structures are identified in the CSD, and allows one to click and see their deformation profiles.

We would interpret this result in the same way that the original authors have, for their normal-coordinate based analysis – where they used a dedicated porphyrin analyser and replaced the N atoms for C, we can look at *Pc* directly. Secondary ligands are shown in their analysis to measurably deform the *Pc* ring by introducing steric demand on the ring and changing electronic demand of the metal centre. Using scsd, we can include *Pc* outer rings in the analysis, though this does not change the

authors’ conclusions; primary ‘ruffle’ with secondary ‘wave’ and ‘saddle’ are also observed here. While the *ruffle* deformation is induced by ancillary ligands, one can assume that the smaller *wave* and *saddle* shapes come about due to how the molecule packs in the crystal structure.

Advantages come that we can immediately contextualise this information with previously determined data at www.kingsbury.id.au/scsd_model_ext/phthalocyanine. The FePc(diPrim)$_2$ is more ruffled than any structure observed prior to the 2022.1 database release and, while no very similar structure has been observed, a previous nonplanar structure marked as most similar (CSD:NODVIO, B_{1u}:6.36 Å, C. Hunt, M. Peterson et al, *J. Am. Chem. Soc.*, **2019**, *141*, 2604-2613, DOI: 10.1021/jacs.8b12899) might be a good comparison point. One might also remark that the saddle-shape of nonplanar phthalocyanines (up to 40 Å) is often more dramatic than observed ruffling. Finally, a sentence may be included in the published work; “the crystal structure exhibited obvious out-of-plane deformation; scsd analysis identified a principal ruffle character of 10.83 Å, exceeding previously identified phthalocyanine ruffling” and citing that previous example, as well as this paper.

The formation of a model is more complicated, and is best performed using the interface at www.kingsbury.id.au/scsd_new_model or by launching a local webserver, or by following the instructions in examples/scsd_tutorial.ipynb. These models are stored in /scsd/scsd_models_user.py; making the model requires atom positions and point-group symmetry to be established; the positions are aligned and symmetrized to the symmetry operations by use of *yield_model* which can then be passed into the *scsd_model* constructor. Further analysis is explained in the tutorial notebook which is available at www.github.com/cjkingsbury/scsd/tree/main/example/notebooks

.

2. Implemented Models

Table S1. Implemented models in scsd; see www.kingsbury.id.au/scsd_model/{model} (e.g., www.kingsbury.id.au/scsd_model/tcnq (simple version), or www.kingsbury.id.au/scsd_model_ext/ttf (complex version, v. slow) or www.kingsbury.id.au/scsd_data/porphycene (pandas DataFrame, .pkl))

Chemical name	Model name	Data base	Notes
porphyrin	porphyrin	Y	See NSD for comparison
chlorin	chlorin	Y	Porphyrinoid – $C_{18}N_4$
porphycene	porphycene	Y	Porphyrinoid – $C_{20}N_4$ [2.0.2.0]
corrphycene	corrphycene	Y	Porphyrinoid – $C_{20}N_4$ [2.1.0.1]
norcorrole	norcorrole	Y	Porphyrinoid – $C_{18}N_4$ [1.0.1.0]
phthalocyanine	phthalocyanine	Y	Porphyrinoid – $C_{32}N_8$
subporphyrin	subporphyrin	Y	Porphyrinoid – $C_{15}N_3$
4,4-difluoro-3°,4°-diaza-4-bora-s-indacene	bodipy	Y	Medicinal fluorophore
Cyclobis(paraquat-*p*-phenylene)	blue_box	Y	Common electroactive host
dibenzazepine	carbamazepine	Y	Only the $C_{14}N$ aromatic unit – study in medicinal polymorphism
xanthene	xanthene	Y	Family of dyes
tetracyano-*p*-quinodimethane	tcnq	Y	Molecular electronics – acceptor, exhibits multiple oxidation states
tetrathiafulvalene	ttf	Y	In molecular electronics – good donor
2,5-dihydroxy-p-benzoquinone	dhbq	Y	Common ligand in coordination polymer synthesis, redox active
triptycene	triptycene	Y	Large framework in molecular engineering
1,4,8,11-tetraazacyclotetradecane	cyclam	Y	Showing limits of technique – scsd does not provide a good parameterization for this system
cucurbit[6]uril	cb6	Y	Host in supramolecular chemistry
cucurbit[8]uril	cb8	Y	Larger host in supramolec. Chem.
Corrole	corrole	Y	Porphyrinoid – $C_{19}N_4$ [1.1.1.0]
porphyrin_pdb	porphyrin_pdb	Y	Porphyrin, but from the PDB dataset – different database and naming system
Betacarotene π-system	bcr_pi_pdb	Y	Showing limits of technique – scsd does not provide a good parameterization for this system
calix[4]arene	calix4	Y	Host in supramolecular chemistry
cyclotricatechylene	ctc	Y	Host in supramolecular chemistry
perylenediimide	perylenediimide	Y	Redox component, acceptor and CPL fluorophore
naphthalenediimide	Naphthalene-diimide	Y	Molecular electronics component, smaller cousin of PDI
pyrene	pyrene	Y	

anthracene	anthracene	Y	
naphthalene	naphthalene	N	
benzoquinone	benzoquinone	N	
dodecatungstate (no anion) ($W_{12}O_{36}$)	keggin	N	W&O exterior only
B-carotene	betacarotene	N	Full molecule
Porphyrin-pyrene-cage $(dpm)_6(pyr)_3$ "nanobelt"	ppc	N	See https://doi.org/10.1021/jacs.2c01605 *J. Am. Chem. Soc.* 2022, 144, 9212–9216
teropyrene	teropyrene	N	
Sessler's hexapyrrole [1.0.0.1.0.0] "Amethyrin"	amethyrin	N	
Amethyrin with pyridine at rings B & E	dipyriamethyrin	N	
Brückner's "Siamese Twin" porphyrin *(Diaza-N-confused hexaphyrin)*	siamesetwin	N	
[*acln*]tetrabenzo-pentacene	Tetrabenzo-pentacene	N	
	pentacene	N	
$Pt_2(P_2O_5)_4$	ptpop	N	Early ultrafast crystallography study
1,3,5,7-tetramethyl-BODIPY	tmbodipy	N	
coronene	coronene	N	
bicyclo[1.1.1]pentane	bcp		Showing high strain reduces conformational flexibility
α,α-dipyrromethene	dpm	N	Common ligand; PDT agent
(D_{2h})metal bis (acetylacetonate)	macac	N	Common metal complex
[adjm]tetrabenzo coronene	tetrabenzo coronene	N	Highly distorted aromatic
	azatriangulene	N	Extended aromatic
	texaphyrin	N	Expanded porphyrinoid
[*adjm*]-*o*-thieno-[gp]-benzocoronene	tetrathieno dibenzocoronene	N	Highly distorted aromatic

3. Test Cases for SCSD Analysis

Below are several examples of how scsd might be used to compare, contrast and correlate molecular distortions across different molecular frameworks. The data and notebooks used for the generation of these parameters are provided on request.

3.1 BODIPY compounds

The BODIPY subunit (4,4-difluoro-4-bora-3a,4a-diaza-*s*-indacene) is an important fluorophore in biological studies; being easily synthesized, stable, modifiable and reliably fluorescent. The BODIPY has, in the ideal, assumption of C_{2v} symmetry, and numerous studies have proposed a variety of internal (distances, angles, torsions) and external (plane-to-plane angles, etc.) coordinate frames and calculations to understand variation within the fluorescent lifetimes. The use of pseudo-normal coordinates allows us to cluster structures; alongside chemical modifications, these should correlate with photophysical attributes.[31]
The total set of bodipy compounds can be accessed by scsd_collection('bodipy'). We can perform a principal component analysis (PCA) on the derived matrices which define the eigenvectors of the symmetry coordinates for the class. This gives us a handle for looking at the concerted movement of atoms; these pseudo-normal modes show obvious clusters of structures in this case, derived from the substitution pattern on the bodipy core. The addition of a 1,3,5,7-tetramethyl substituent pattern increases quantum yield; this substituent pattern also shows a distinct structural cluster in $A_1(1)$ and $A_1(2)$ (see Fig. 4 in main paper). Cluster identification is described in *ellipsoid.py*.

3.2 Anthracene

Anthracene is a polycyclic aromatic hydrocarbon ($C_{14}H_{10}$) with D_{2h} symmetry; here, we're looking at all molecules with the anthracene substructure of three fused rings. Anthracenes are formed with – and have been structurally characterized with – variable substitution patterns and electronic modifications. The related anthraquinones (anthracene-9,10-dione) are commonly used as dyes in nature and industry.
The central anthracene core is often depicted as a regular structure, and data confirms that examples are often minimally distorted from planarity. Introduction of significant steric bulk of substituents in 'decasubstituted' examples (e.g., 1,2,3,4,5,6,7,8,9,10-decaphenylanthracene) allows for the activation of a nonplanar distortion mode – notably the A_u mode (4.61 Å) shown in interactive Figure S1 (https://www.kingsbury.id.au/scsd/ZOPJUJ).

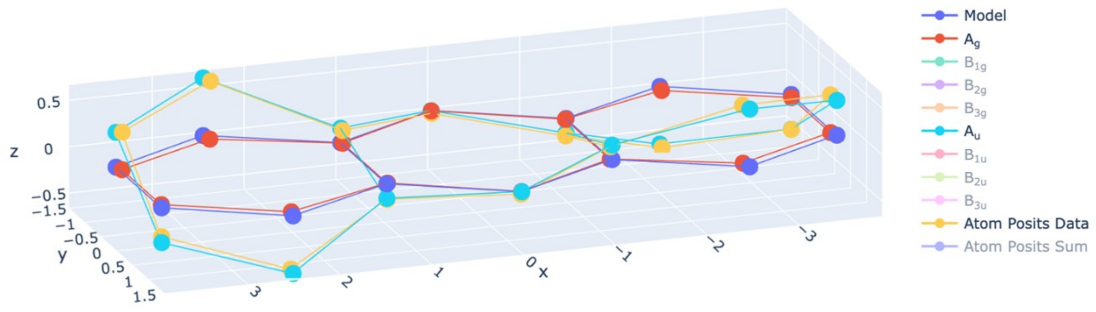

Figure S1. The A_g (red) and A_u (periwinkle) distortions of the model anthracene (blue) towards the twistacene (CCDC: ZOPJUJ).[32]

Strapped systems, such as the anthracene dimeric cyclophane bi(anthracene-9,10-dimethylene), show an entirely different distortion along the B_{1u} mode (yellow, 2.44 Å) (Fig. S2).

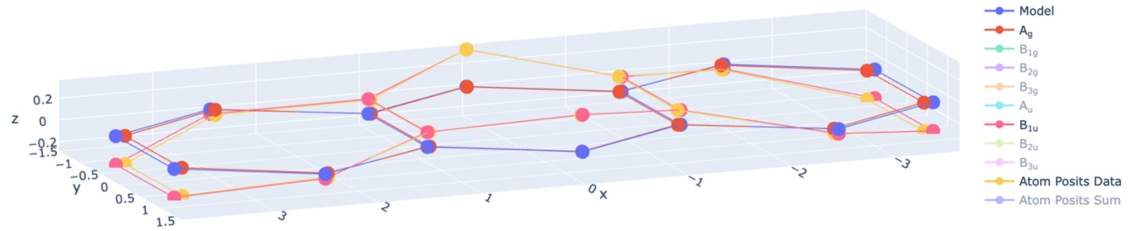

Figure S2. The A_g (red) and B_{1u} (pink) distortions of the model anthracene (blue) towards the measured conformation of the cyclophane component (yellow, CCDC: ANTMEU03).[33a]

This system was used as a model for the 4+4 cyclization of these anthracene systems. As we can see, upon the progression of this reaction to completion (i.e., to 9,9':10,10'-diethano-9,9':10,10'-bi-9,10-dihydroanthracene) the increase of the 'v' shape – and corresponding large increase in the B_{1u} value (to 6.59 Å, MOFGAQ) (Fig. S3).

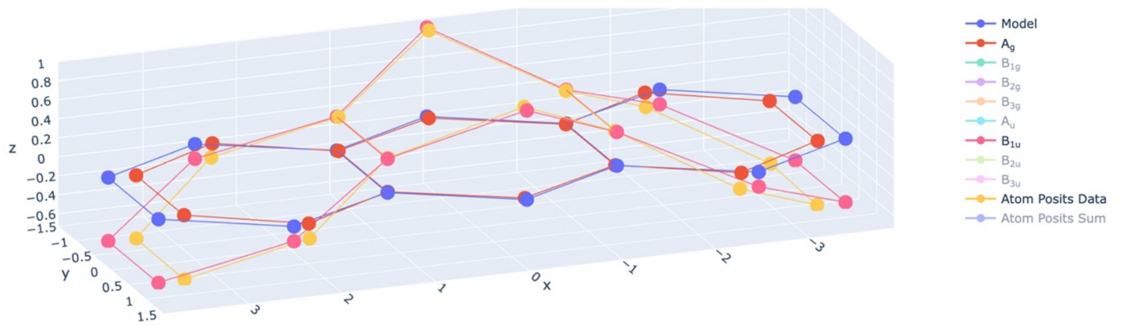

Figure S3. SCSD analysis of the anthracene core of CCDC: MOFGAQ, demonstrating the B_{1u} and A_g deformation from dimerization of the parent 9,10-diethoxyanthracene. The summation of the model (blue) with these two deformations (red and pink) and other minor components results in the atom positions (yellow).[33a]

When reacting the anthracene core to form the endoperoxide, a similar value is achieved (6.98 Å) (Fig. S4).

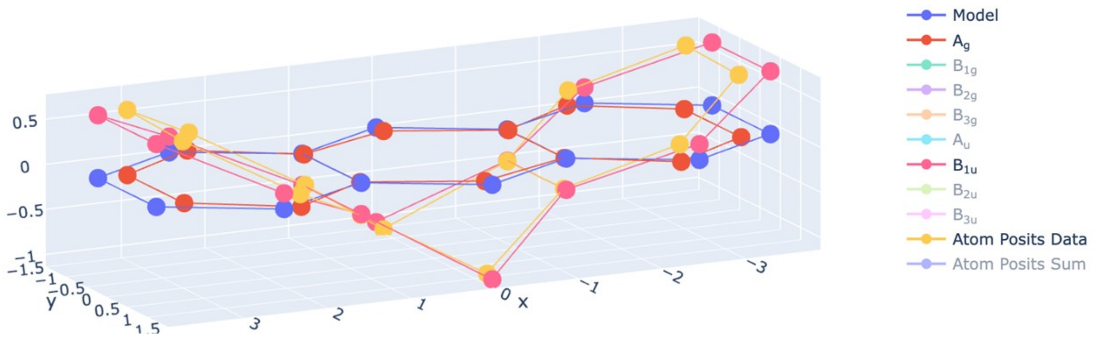

Figure S4. SCSD analysis of the anthracene core of CCDC: CICWEQ, the B_{1u} principal deformation from formation of an anthracene-9,10-endoperoxide is largest.[33b]

The inclusion of both of these motifs – *i.e.* a sterically hindered anthracene endoperoxide, showed both of these motifs simultaneously. (A_u:1.52 Å, B_{1u}:7.12 Å, WUHTUO) (Fig. S5). A_u deformation is similar to other crowded anthracenes, and B_{1u} deformation is approximately equal to that for the model anthracene-9,10-

endoperoxide. This demonstrates the power of a simple additive process that is remarkably accurate at predicting dissymmetric deformations, and therefore measured atom positions.

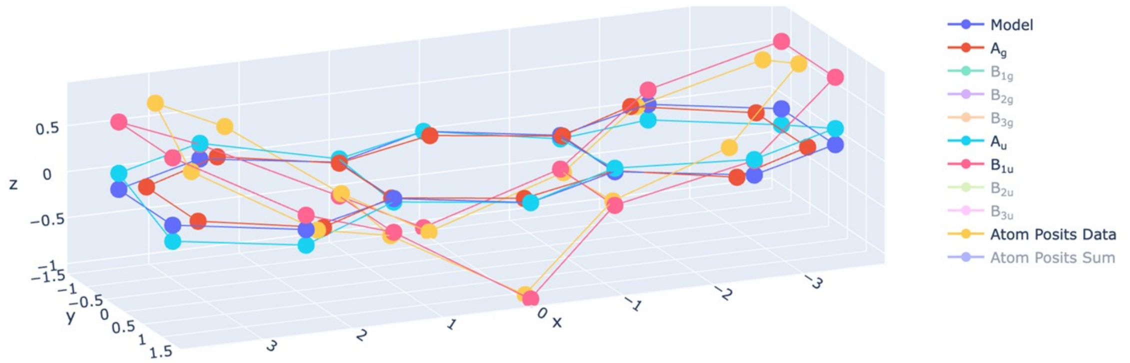

Figure S5. SCSD analysis of the central anthracene core of the tetrabenzopentacene derivative in CCDC: WUHTUO.[33c]

These are, of course, among the most distorted examples, and compounds 1.3-1.5 have a non-anthracene electronic core, so would present non-anthracenoid photochemistry. From this data, we can however preview the modes of distortion applicable to a small unit may be inherited when composing part of a larger molecule, giving some indication of local curvature in an extended system. This approach is being investigated.

3.3 Tetrathieno-dibenzocoronenes

For model used see:
https://www.kingsbury.id.au/scsd_model/tetrathienodibenzocoronene

Chiu *et al.* described a series of coronenes which, as a function of the clash of the external substituent benzo- and thieno- groups form two different conformations.[34] These molecular systems were described as ‘contorted’ – and show a clear case for the conformational typing available using SCSD. The four crystal structures are described using a series of plane-to-plane angles and variable descriptions (“up-down”, e.g., Fig. S6 and “butterfly” (Fig. S7, UVOMUO) which can be simply extracted as the eigenmodes of B_{3g} for “up-down” and B_{1u} for “butterfly”. The values of these deformations can be tabulated (B_{3g}: 18.58 Å for UVOMOI, 18.38 Å for UVOMIC and 21.33 Å for UVONAV; B_{1u}: 25.78 for UVOMUO). The increased value for UVONAV reflects the influence of the C_{60} supramolecular adduct in UVONAV on the molecules’ shape.

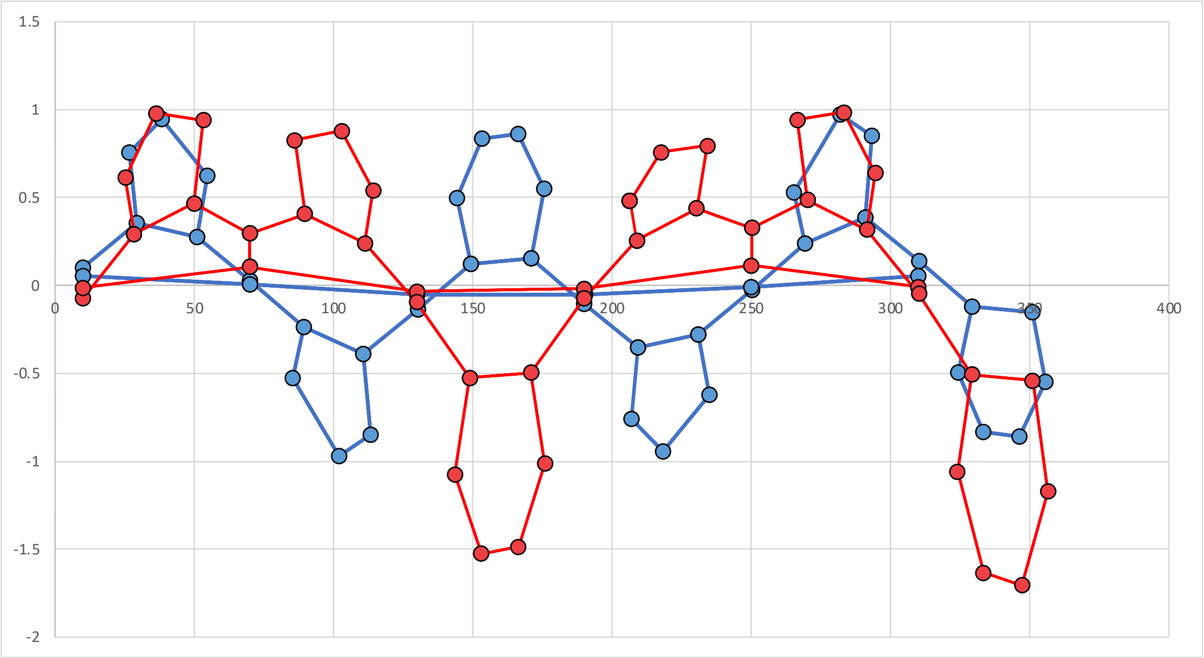

Figure S6. Skeletal deviation plot for UVOMIC (blue) and UVONAV (red) showing the two different conformational profiles. Deviation table values are in the hidden box at the bottom of the standard scsd output and can be copied to an external plotting program.[34]

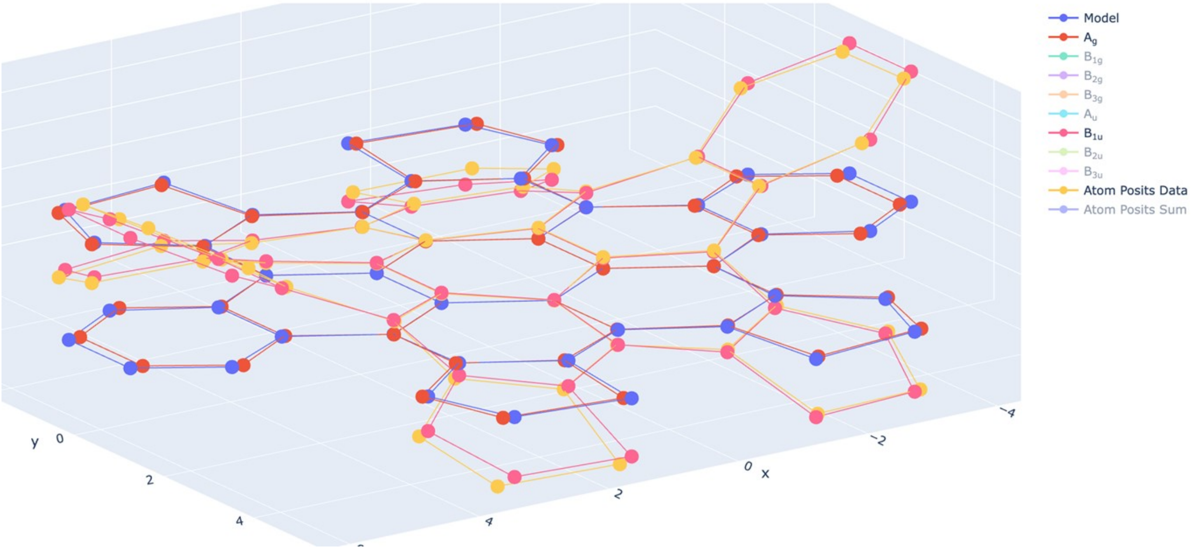

Figure S7. SCSD plot of the butterfly deformation of CCDC:UVOMUO; the molecule deforms along its B_{1g} dissymmetric mode, resulting in C_{2v} symmetry .[34]

This parameterization also makes the identification of the pertinent point groups trivial by inspection of the Mondrian diagram. Finally, good agreement is found between the measured eigenmodes of each example, even with drastically different magnitudes (i.e., B_{3g} in UVOMUO at 1.66 Å).

3.4 The “Blue Box”

For model used see: www.kingsbury.id.au/scsd_model/blue_box

3.5 Cucurbituril CB[6] – supramolecular host

For model used, see: https://www.kingsbury.id.au/scsd_model/cb6

CB[6] is a mainstay of supramolecular chemistry – the simple host is easily formed and commercially available, and has many crystallographically identified examples. Linked by single bonds, successive examples of the cucurbituril series display increasing conformational flexibility which has not been rigorously explored. As such, CBs are a useful example to demonstrate the role of host deformation resulting from host-guest interactions.
In the ideal setting, CB[6] conforms to a D_{6h} point group. Point group D_{6h} includes what we will call ambiguous E-groups – the number of independent orientations of some symmetry operations (3) exceeds the identity of those symmetry operations from the point group table (E = 2). As such, the program 'orders' the representations such that the highest symmetry in any setting is reproduced reliably. The treatment of clustering with these groups is nontrivial, where a particular motif (E_{2g} ovoid deformation, for example) may be represented in 3 different parameters (E_{2g}1, E_{2g}2, E_{2g}3) aligned to the 3 equivalent D_{2h} subgroups. Work on presenting these parameters with cylindrical systems (e.g., Cremer-Pople coordinate systems) is ongoing.
Single monomer inversion (Fig. S8) can be seen in the A_{1g} decomposition result, with the regularly oriented cucurbiturils forming a single cluster in the lower right. Looking closer at this cluster, we can see that the encapsulated compound has a large effect on isotropic expansion of the host. Compounds containing the R-NH(CH_2)NH-R unit are contracted, and those with R-(CH2)$_6$-R inside are expanded (Fig. S9).

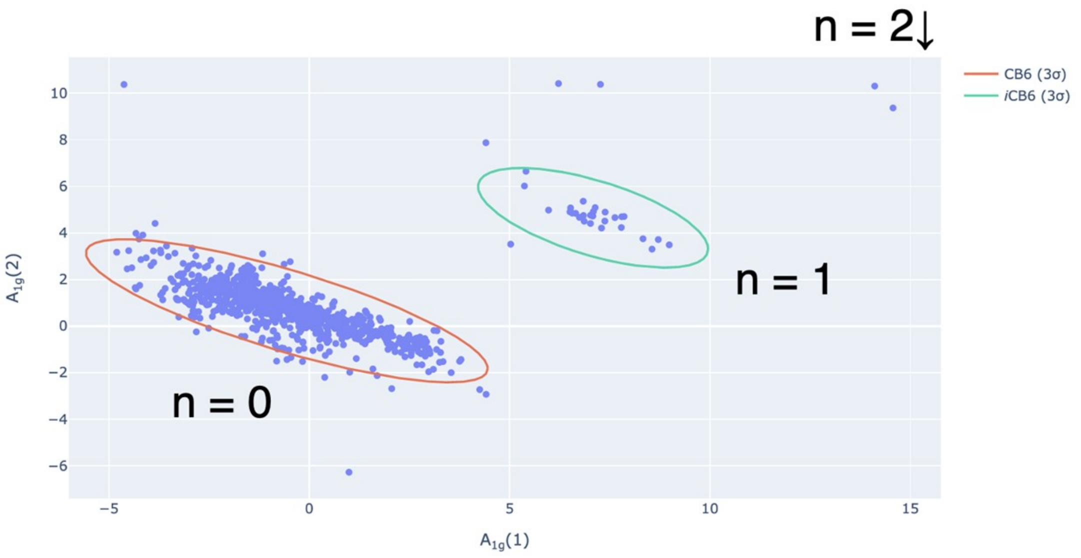

Figure S8. A_{1g} distortion in CB[6], where *n* refers to the number of inverted units (H- atoms facing inwards).

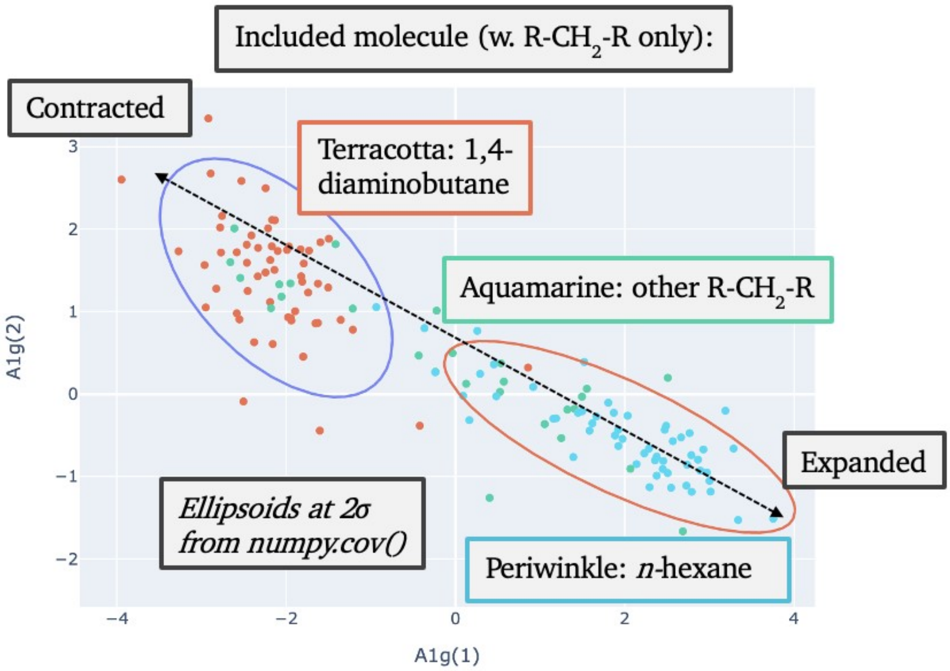

Figure S9. A_{1g} distortion in CB[6] main cluster, highlighting the movement attributable to inclusion of different types of interstitial guests.

3.6 Nanotube fragments – cycloparaphenylenes

The SCSD analysis technique can be adapted to characterizing the symmetry concerns of ultrashort nanotubes, compounds such as cycloparaphenylenes (CPP) which,[37] while not what one would think of as 'rigid groups' are rigidified by annular strain. Nanotubes have been simulated which exhibit morphological changes, but only a few crystallized examples of low-MW oligomers are available. We have included exemplar analyses of [6]CPP (~D_{6h}), octabenzo-[4]CPP (~D_{4h}) and cyclo-2,7-tetra(4,5,9,10-tetrahydropyrene) (i.e., octaethylene-[8]CPP, ~D_{4h}) (Fig. S10). In each case, we can directly observe the prominence of the alternating aryl-aryl non-coplanarity which deform the solid-state structure, and some level of ovaloid squashing, but these take on different symmetry characters in each case. In [4], this is by B_{2u} (to D_{2h}) for the rotation, and B_{1g} for the (barely observed) squash. In [6] this is B_{1g} (to D_{3d}) for the alternation, and no squash is observed, as this compound (in LAXKOM)[37d] lies around an S_6 axis in R -3. In [8] (ZOFQUI),[37e] the two motifs can be assigned as the $E_g(x+y)$ (aryl-alternation) and B_{1g} (squash), respectively, and both are prominent.

The deformation experienced by an archetypal ring is obviously going to vary between crystal structure measurements, especially when these molecules include guests. This SCSD method is a simple way of comparing and contrasting these units, even between rings of different sizes.

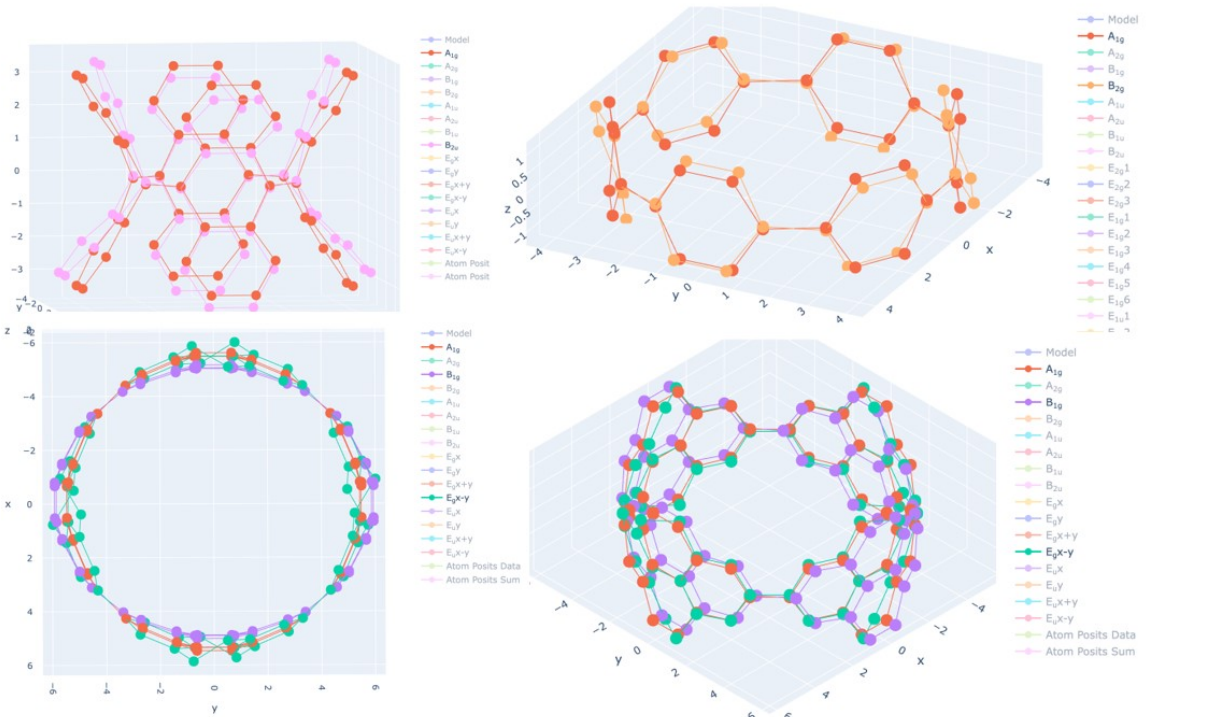

Figure S10. Prominent SCSD deformation modes for the compounds (a) octabenzo-[4]-paracyclophane (b) [6]-paracyclophane (c-d) octaethylene-[8]-paracyclophane.

3.7 Perylene-3,4,9,10-tetracarboxylic diimide

For full details of models see: https://www.kingsbury.id.au/scsd_model_ext/perylenediimide

Assigning structural variation across a database
Perylene-3,4,9,10-tetracarboxylic diimide (PDI) materials are highly interesting as electronic components and sensors, with strong absorption and luminescence, good electron donor properties and facile synthesis from amines and perylenetetracarboxylic dianhydride.[38]
Contorted conformations of the polycyclic core can be accessed by varying the patterning around the free 1,2,5,6,7,8,11,12- positions or the perylene component. The 319 crystal structures of PDI with identified coordinates were analyzed with SCSD (Fig. S11).

Generally, these compounds can be divided into four groups – the 'twisted' A_u (substitution at 1,6,7,12; higher sum VdW = higher distortion); 'bent' B_{1u} (2-12 & 5-11 strapping, 1,7- sub pattern or supramolecular adduct); 'wavy' B_{2g} (C_2 on x) or B_{3g} (C_2 on y) (from substitution at 2,5,8,11 pos.) and the 'pure' A_g contraction, from fusion of 1-12 and 6-7 positions into *e.g.* thiophene-like ring. Some structures (e.g., BAMBUO) contain multiple motifs towards dissymmetry.

Distorted PDI compounds are particularly interesting as circularly polarized luminescent (CPL) materials and, as we can demonstrate here, the only meaningful pathway towards a CPL-compatible point group (C_1, C_2, D_2) is via A_u distortion. We

expect that the CPL behavior will correlate with this measured value in PDIs, and that generally luminescent molecules with high chirality-inducing SCSD values will elicit interest as CPL materials.

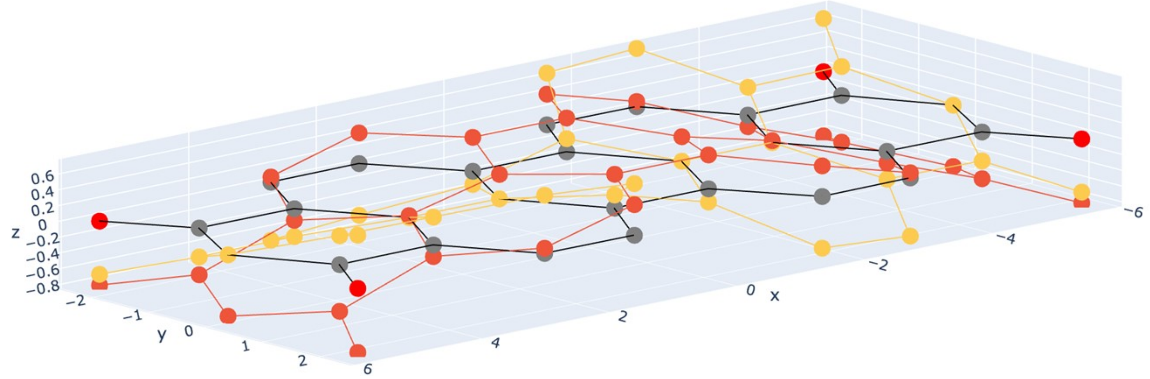

Figure S11. The perylenediimide model, in gray overlaid with the $A_u(1)$ (yellow) and $B_{1u}(1)$ (red) modes calculated by principal component analysis of the 319 examples.

3.8 Dipyridyldicarbazole

For full details of models see: www.kingsbury.id.au/scsd_model/dipyridyldicarbazole

A recent article by Rickhaus and coworkers[39b] discusses the effect that a saddle-shaped macrocycle has on a directed self-assembly and uses the example of substituted dipyridyldicarbazoles macrocycles developed by Müllen's group[39a] (Fig. S12). The saddle shape can be termed B_{1u} distortion in SCSD; of 25.8 Å for $Co^{II}(tBu_4dpdc)$ and 26.8 Å for $H_2(tBu_4dpdc)$ from the crystallographic determinations previously reported (EGICAC and EGICEG). Other out-of-plane distortions (A_u, B_{2g}, B_{3g}) hold small, non-zero values.

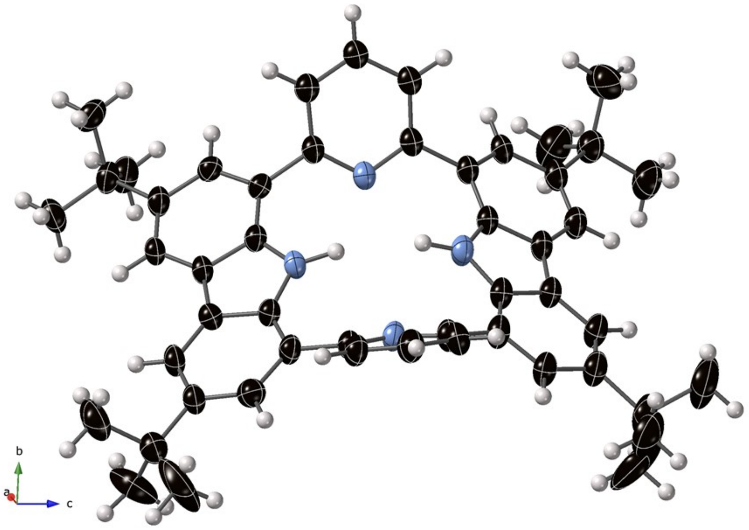

Figure S12. The saddle-shaped molecule tetrakis(*tert*-butyl)cyclo-dipyridyldicarbazole (CCDC: EJICEG).[39a]

The 'saddle' shape here is a critical factor in how these molecules assemble into oligomers for *n*Hx_4dpdc; a single term can be used in place of multiple bond torsion factors to compare saddle character. Calculation of a comparative model is not needed to run SCSD, and this can simplify the process of comparing several chemical motifs.

As such, other molecular units with a saddle character can be parameterized in this same way, with the understanding that the saddle-induced stacking supramolecular arguments made in Rickhaus' paper[39b] can be extended to identify other molecules with an appropriate shape.

3.9 – A selection of porphyrinoids

The structural versatility of polyheterocyclic macrocycles is vast; with SCSD, we can start to understand the conformational variance across and between distinct examples within the class. Porphyrin molecules were scrutinized across the chemical literature in our previous paper[12] using the normal-coordinate structural decomposition method (NSD) with a focus on how the properties of the macrocyclic chelate may be modified by chemical alteration. This program is available at www.sengegroup.eu/nsd for user samples. Krumsieck and Bröring expanded this methodology to a selection of common pyrrolic macrocycles (corrole, norcorrole, porphycene, corrphycene) which are available to be analyzed through PorphyStruct.[11] New porphyrin-related compounds are being identified constantly, and a method to analyze the conformation of novel examples is useful. The NSD method demonstrated to us the interplay between macrocycle conformation and coordination environment is a critical factor in designing the shape of chelates and catalysts, and their resultant chemical function. Expanding the scope to greater numbers of related materials can hopefully translate these principles to a broader class of materials.
Exemplary cases from prominent researchers of the expanded porphyrin zoo are included below.
Sessler and coworkers have explored the chemistry of many expanded porphyrins, among those amethyrin (model: 'amethyrin') which contains 6 pyrrole rings as two methine-bridged tripyrrole 'halves' and D_{2h} ideal symmetry.[40] The structure of the protonated form (NENBIR)[40b] is obviously non-planar – distorting by B_{2g}, or 'monkey-saddle' C_{2h} shape, at 9.5 Å. A Zn complex of a similar amethyrin (SURXIM)[40a] is only distorted 7 Å, whereas the Cu_2Cl_2 chelate of this ligand (ZUBGEI)[40c] is distorted by A_u, B_{1u} and B_{1g} to approx. 10 Å each, resulting in a near-C_2 twisted-and-bent shape. A related dithia-macrocycle (TOKYUP)[40d] is approximately planar. The related dipyriamethyrin (with two pyridines in place of pyrrole, model: "dipyriamethyrin", D_{2h}) displays this same twisted-and-bent shape, further exaggerated (Fig. S13, with UO_2, TOZGIB,[40e] to 25 Å A_u and 19 Å B_{1u}).

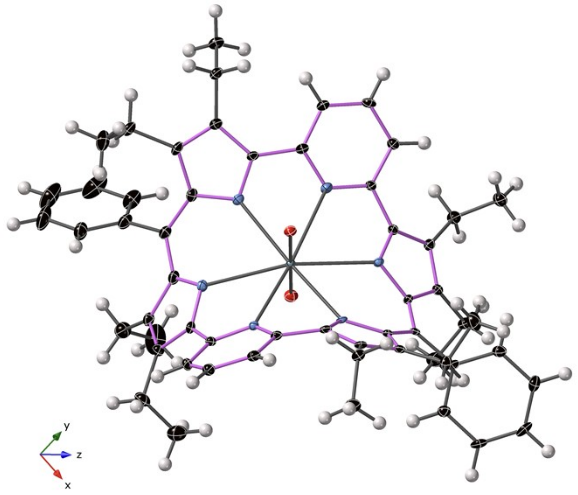

Figure S13. The dipyriamethyrin-uranyl complex (CCDC: TOZGIB).[40e]

Brückner, Meyer and coworkers have explored the "Siamese-twin" diporphyrins (model: "siamesetwin") in their ability to bind two metals, and for their remarkable "twisted" character.[41] This *twisted* character can be named (A_u) and given a value (44.3 Å for the Cu-chelate DOFRIB)[41b] to compare with the Pd_2 equivalent structure (VASKUY, 42.5 Å A_u + 3.3 Å B_{1u} *bent*)[41c] (Fig. S14).

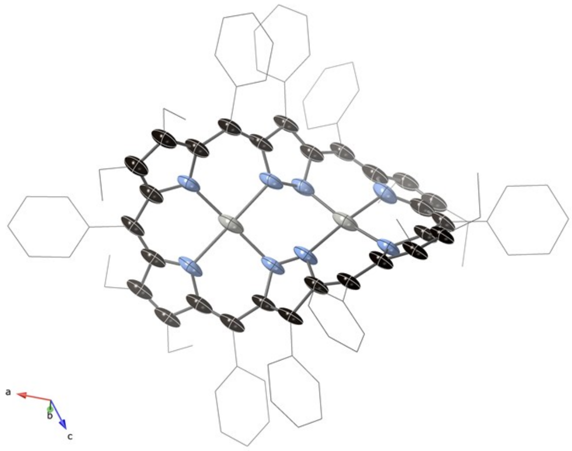

Figure S14. The crystal structure of the highly-substituted Pd_2 diporphyrin (CCDC:VASKUY).[41c]

Kobayashi and coworkers have investigated several aspects of nonplanar phthalocyanines.[42] The series $(ChPh)_8$-pc (Ch = S, Se, Te) show an interesting case of substitution change inducing changed photochemical properties through structural distortion. The *saddle* structures (S: UQALEF, Se: UQAKUU, Te: UQALAB) increase in B_{2u} (18.5 – 24.3 – 42.5 Å) with a big jump for the Te substituents, and a corresponding large change in the characteristic photochemistry.
A triply-fused diporphyrin (available as *triply-fused-diporphyrin*) from Osuka's group was characterized as the Cu and Zn chelates – both approximately planar.[43] We can see the expansion of the core in the Zn example; in both, a *tilda* ("~", B_{3g}) and double-twist (B_{2g}) motifs are present, resembling the Lissajous curves (a=1, b=3, f = 0 and π/2).

Gros, Paolesse, Gross and others have been instrumental in exploration of the chemistry of corroles.[44] With a core similar to vitamin B12 (a corrin, or decahydrocorrole), the corrole system is particularly interesting for the formation of first-row transition metal complexes with a constrained coordination environment as compared to porphyrin. A full database is available at https://www.kingsbury.id.au/scsd_model/corrole to investigate structure-function relationships in the photophysical and reactivity of these systems. These insights are especially interesting now that corroles are being included into heme-vacated enzyme pockets – designing protein-induced conformational change is an important contemporary research area.[27c,45] Corroles are possible to analyze through *PorphyStruct (see comment above)*– larger constructs, such as the biscorrole (VATTOC),[44b] can be analyzed by the SCSD method – showing a C_i conformation of the global π system resulting from core proton repulsion.

3.10 Teropyreneophanes

For model and details see: www.kingsbury.id.au/scsd_model/teropyrene

A series of model cyclophanes, the teropyreneophanes, were synthesized by intricate chemistry by Bodwell and coworkers.[46] Several examples of systems of type **11** with 6-9 C-atom apical bridges were crystallographically identified [x = 4, CCDC: JUWSOM;[46b] x = 3, QEYCOO;[46c] x = 2, GUHSEI;[46d] x = 1, NOKJAB[46a]] and their SCSD values are shown in Table S2.

Table S2. SCSD values for the series of teropyreneophanes.

	JUWSOM	QEYCOO	GUHSEI_1	GUHSEI_2	NOKJAB
	x = 4	x = 3	x = 2	x = 2	x = 1
**Symm.**	**Sum (Å)**				
A_g	28.36	33.61	37.01	36.00	41.93
B_{1g}	1.30	1.05	0.48	3.09	1.26
B_{2g}	0.22	0.26	1.33	0.37	0.23
B_{3g}	0.87	0.28	0.19	0.21	0.09
A_u	1.27	0.93	0.36	2.54	0.93
B_{1u}	48.57	51.42	54.01	53.36	56.52
B_{2u}	1.24	0.67	0.26	0.33	0.22
B_{3u}	0.23	0.50	2.32	0.82	0.43

From this data, we can observe the shape effects of decreasing strap length. The increase in out-of-plane distortion along B_{1u} and the corresponding in-plane A_g distortion with decreasing strap length is approximately linear, allowing for extrapolation of the obtained values – for example, the x = 0 example (a -$CMe_2(CH_2)_4CMe_2$- strap, hypothetical) can be expected to hold values of A_g: 44 - 46 Å and B_{1u}: 58.4 - 59.6 Å with this linear approximation (Fig. S15).

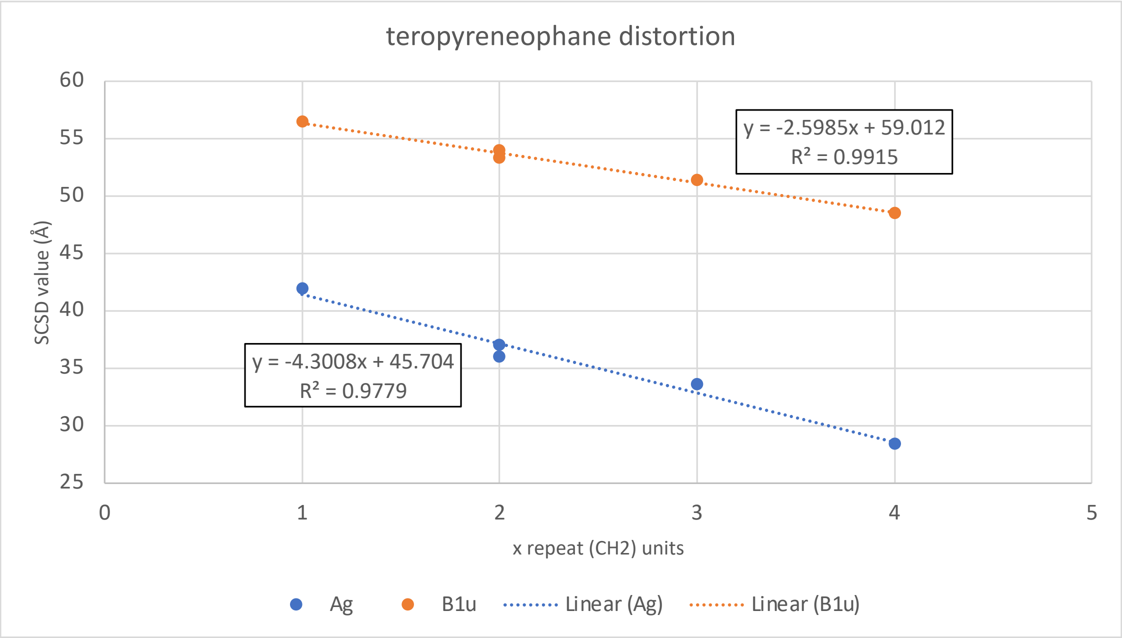

Figure S15. Linear fits to SCSD values across the strapped teropyrenes.

Further distortion from the parent C_{2v} shape can be catalogued as two types – the double skew of B_{2g} + B_{3u} (B_2 in C_{2v}) to C_s, and the twist of B_{1g} + A_u (A_2 in C_{2v}) to C_2. These are present to varying degrees by each of the examples, more pronounced when x is even, and demonstrated by the L-shaped block in the relevant *Mondrian* diagrams, such as the purple area in Fig. S17.

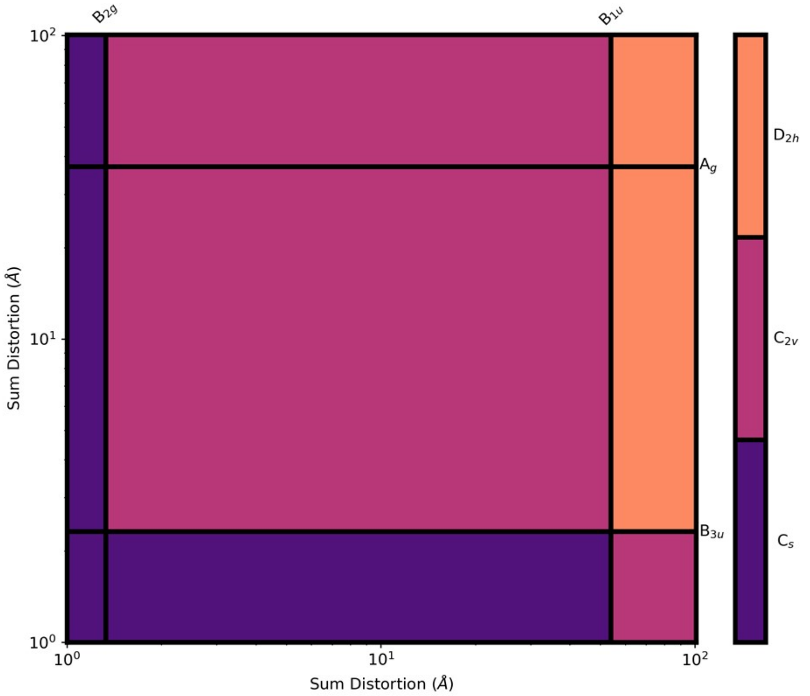

Figure S16. Mondrian diagram for the teropyrene component of 2,9-dimethyl-2,9-decanyl-teropyrenophane (CCDC: GUHSEI,[46d] molec. 1).

From this data we can make reasonable assumptions about the likely shape of molecules in the series, and potentially about apically strapped polyaromatics generally – that major B_{1u} *bending* distortion is approximately linear with strap length, and two types of minor distortional affect are present, but not generally simultaneously, depending on whether the $(CH_2)_n$ chain repeat number n is even (A_2) or odd (B_2).

3.11 Time-resolved crystallography – [Cu(I)(dmp)(dppe)][PF_6]

An interesting case study for SCSD parameterization presents in molecules undergoing changes in their bonding. A HOMO-to-LUMO excitation should result in changes to the bonding strength and overall symmetry of a hypothetical π- system. The simplest example would be ethene – excitation π→π* would reduce the strength of the bond, and relaxing the planarizing influence of this bond, as anti-bonding is now present. Therefore, upon excitation, we could expect alteration in A_g and A_u SCSD structural parameters (in D_{2h}). This molecule is not ideal for this experiment for many reasons – with analysis reliant on H-atom positions, and the lifetime of ethene excitation approx. 50fs,[47a] which is shorter than X-ray free electron laser (XFEL) experiments can resolve. Much better candidates for studying this behavior exist, such as the two examples of pump-probe experiments discussed below.

The [Cu(I)(dmp)(dppe)][PF_6] (dmp = 2,9-dimethyl-1,10-phenanthroline; dppe = 1,2-bis(diphenylphosphino)ethane) was measured in ground and excited state structures – with no excitation (off) and 0-50μs after excitation with 355nm light (on).[47b]
The parameterization of the derived atom positions allows us to see that, upon excitation, A_2 (aileron) and B_2 (flap) asymmetric distortions change in both of the independent molecules, by around -10% (Table S3). We are expecting to see an MLCT excitation, (Cu^{I} → Cu^{II} and dmp → dmp^{-}) – with electrons transferred to the π-system increasing planarity. As indicated in the paper by Vorontsov *et al.*, there was less change in the ligand than was expected from parallel theoretical investigations. Molecular shape of these two independent molecules within the crystal structure is reminiscent of that observed for [Cu^{I}(phen)(PPh_3)$_2$] (phen = 10-phenantroline-N,N) with its photophysical bimodality induced by shape differences.[47c]

Table S3. SCSD parameters for the dmp component in [Cu(I)(dmp)(dppe)][PF_6] in both the light-on and light-off states.

Light (Molec.)	Off (1)	On (1)	Off (2)	On (2)
**Symm.**	**Sum (Å)**	**Sum (Å)**	**Sum (Å)**	**Sum (Å)**
A_1	0.00	0.08	0.28	0.25
A_2	0.42	0.37	0.67	0.62
B_1	0.36	0.34	0.64	0.61
B_2	1.05	0.96	1.67	1.50

The [Pt_2($H_{2.5}$pyrophosphite)$_4$] molecule (model “ptpop”, https://doi.org/10.1246/bcsj.77.933) is another system in which the excited state structure has been investigated.[48d] The structure has near D_{4h} symmetry, which approximates a D_{2h} conformation due to bending. The A_{1g} deformation (contraction of the Pt-Pt distance and Pt-P distances) was highlighted by the authors; a corresponding B_{2g} distortion can be observed (Table S4).

Table S4. SCSD parameters for the $[Pt_2(H_{2.5}P_2O_5)_4]^{2-}$ anionic complexes in the crystal structures of two $(Bu_4N)_2[Pt_2(H_{2.5}P_2O_5)_4]$ polymorphs in the irradiated and non-irradiated states (XUVNAD-XUVNAD03).

	XUVNAD	XUVNAD01	XUVNAD02	XUVNAD03
**Polymorph**	**Bu2**	**Bu2**	**Bu1**	**Bu1**
**Light**	**off**	**on**	**off**	**on**
**Symm**	**Sum (Å)**	**Sum (Å)**	**Sum (Å)**	**Sum (Å)**
A_{1g}	0.00	0.07	0.50	0.31
A_{2g}	0.37	0.39	0.38	0.40
B_{1g}	0.34	0.31	0.35	0.33
B_{2g}	7.25	7.31	6.98	6.93
A_{1u}	0.08	0.05	0.00	0.00
A_{2u}	0.08	0.03	0.00	0.00
B_{1u}	0.06	0.06	0.00	0.00
B_{2u}	0.05	0.07	0.00	0.00
E_gx	1.88	0.66	1.82	1.79
E_gy	0.64	1.89	0.65	0.65

E_ux+y	0.15	0.09	0.00	0.00
E_ux-y	0.11	0.17	0.00	0.00

The B_{1g} distortion measures several atoms and can therefore be more telling of structural change than an individual distance. While the Pt-Pt distance changes by 0.0081 – 0.0127 Å, the corresponding movement of the other 28 atoms totals +0.04 Å – so easier to measure. This can be likened to observing the mechanical advantage of a drill-press, and the concerted movement of multiple atoms – independent of minor distortions. Of course, this is dependent strongly on the polymorph structure – the “Bu2” polymorph (XUVNAD and XUVNAD01) exhibits much stronger B_{2g} distortion than “Bu1” polymorph (XUVNAD02 (off) and XUVNAD03 (on)) by 0.3 Å (6.97 –> 7.31 Å) – though shows similar change on excitation (+0.07 Å). Each structure exhibits $E_g(x)$ deformational character of around 1.8 Å.

This intricate analysis of the ligand conformation is difficult to perform when considering only internal coordinates, and SCSD allows easy depiction of this deformation. Similarly, the concerted nature of coordinate extraction is a stopgap against random errors – the movements are essentially all measured multiple times in orthogonal directions.

4. Mondrian Typing

Mondrian graphs[15] are often of a few distinct types (Fig. S17). This is a short list of commonly encountered examples.

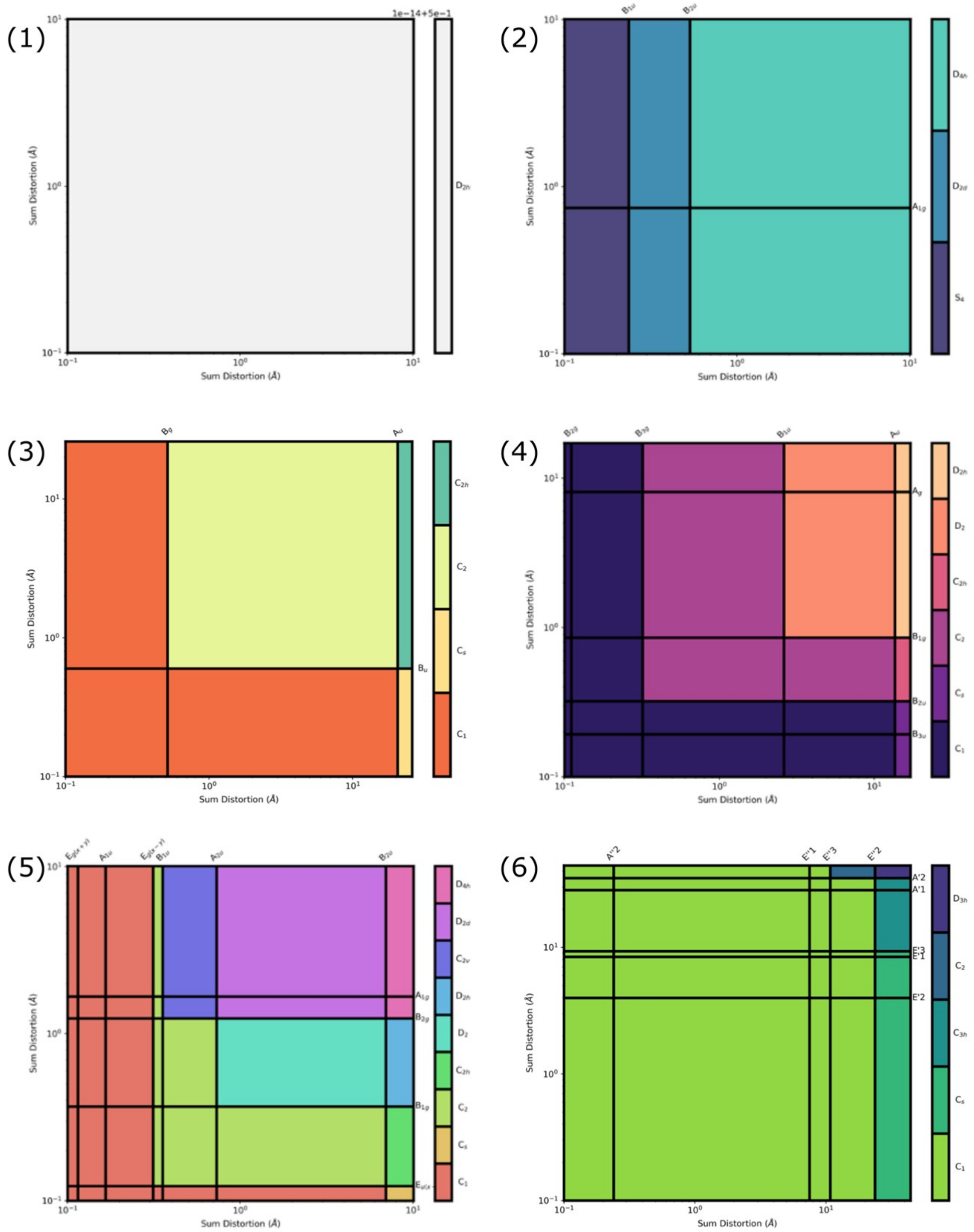

Figure S17. Six common types of Mondrian graph, types 1-6 below. Color schemes are at the discretion of the user; a selection of schemes (Greys, mako, Spectral, magma, hls, viridis_r) are shown here.

Type 1. Approximately symmetric
(a) NAPHTA16[49a] – www.kingsbury.id.au/scsd/naphta16
No deviation from symmetry above 0.1 Å sum; the parent molecule is very close to the ideal point group symmetry in this case, but may also be perfectly symmetric, if the atoms lie on special positions or have been held to constraints, e.g., with AFIX 116 for naphthalene. The Mondrian diagram demonstrates close-to-perfect symmetry (and is itself symmetric).

Type 2; Minimally distorted
(b) ZNTPOR06[49b] - www.kingsbury.id.au/scsd/zntpor06
This measurement of [ZnTPP(H_2O)$_2$] in *I*-4*c*2 is nearly planar - some minor out-of-plane distortion is present, indicating S_4 symmetry. An additional A_{2g} commensurate distortion is just below the usual threshold of significance (0.07 Å). Comparing with ZNTPOR02[49c] in *I* 4/*m* which shows a *Type 1* pattern – resulting from the mirror plane of the space group.

Type 3; Large principal distortion with little extra movement
AKUSIK[49d] – dibenzo-(1,9),(5,11)-bis(acenaphtha)tetracene – www.kingsbury.id.au/AKUSIK.html
This diagram represents a compound which is strongly deformed in one dimension (i.e., the A_u of C_{2h}, with a huge gap (approx. 2 orders of magnitude) to the other distortions. In this occurrence, the molecule's C_2 symmetry is still only approximate, but it would make little sense to refer to it by any other point-group. This is demonstrated by the C_2 block which occupies much of the upper right quadrant.

Type 4; Large principal distortion with progression of modes
JEZXOE[13] – analysis of the isolated pentacene core – www.kingsbury.id.au/JEZXOE.html
One large distortion (A_u, twisting of the pentacene) can be observed in the top right corner as the principal distortion, followed by A_g, B_{1u} (and B_{1u} x A_u = B_{1g}) and B_{3g} (and corresponding B_{2u}). The L-shaped blocks appear because, in D_2, B_{1u} and B_{1g} both transform as B_1, retaining the C_2(z) operation, and thus are degenerate. Similarly, in C_2, B_{3g} and B_{2u} each transform as B, finally settling on C_1.

Type 5; Emergent
TUSSIM[27d] – https://www.kingsbury.id.au/scsd/tussim
This molecule was the focus of trying to design a chirality-responsive porphyrin – using two distortion-inducing substitution patterns (a *saddle* (core protonation) and *meso-stretch* (from 5,15-substitution)), the D_2 symmetry (turquoise) achieved by their combination is demonstrated handily by this graph. As such, "Emergent" graphs occur when the two major elements of distortion provide orthogonal dissymmetry.

Type 6 – Error
A misattribution of the atoms to the reference, using the wrong model or the wrong point group may result in a Mondrian diagram that is in error, and looks something like this – clustered lines in the upper right corner. This example (CSD: EJICAC),[39a] was analyzed with D_{3h} symmetry in place of the intended D_{2h} – thus a meaningless result. Care needs to be taken when looking at highly branched molecules, or those with many atoms, that assignment is correctly performed. If this high degree of non-

hierarchical distortion is present in a correctly assigned structure, it may be a sign that this molecular system is not ideal for SCSD analysis or requires a stronger assignment method, such as by-graph alongside an undistorted model.
